# Object segmentation controls image reconstruction from natural scenes

**DOI:** 10.1371/journal.pbio.1002611

**Published:** 2017-08-21

**Authors:** Peter Neri

**Affiliations:** Laboratoire des Systèmes Perceptifs, Département d'études cognitives, Ecole Normale Supérieure, PSL Research University, CNRS, Paris, France; McGill University, Canada

## Abstract

The structure of the physical world projects images onto our eyes. However, those images are often poorly representative of environmental structure: well-defined boundaries within the eye may correspond to irrelevant features of the physical world, while critical features of the physical world may be nearly invisible at the retinal projection. The challenge for the visual cortex is to sort these two types of features according to their utility in ultimately reconstructing percepts and interpreting the constituents of the scene. We describe a novel paradigm that enabled us to selectively evaluate the relative role played by these two feature classes in signal reconstruction from corrupted images. Our measurements demonstrate that this process is quickly dominated by the inferred structure of the environment, and only minimally controlled by variations of raw image content. The inferential mechanism is spatially global and its impact on early visual cortex is fast. Furthermore, it retunes local visual processing for more efficient feature extraction without altering the intrinsic transduction noise. The basic properties of this process can be partially captured by a combination of small-scale circuit models and large-scale network architectures. Taken together, our results challenge compartmentalized notions of bottom-up/top-down perception and suggest instead that these two modes are best viewed as an integrated perceptual mechanism.

## Author summary

Biological vision is designed to discover the structure of the environment around us. To do this, it relies on ambiguous and often misleading information from the eyes: the boundary of a critical object may be invisible against a background of similar appearance, and may be overlooked in favour of the sharp contour projected by an irrelevant shadow. It remains unclear how human vision sorts different image features according to their relevance to the layout of objects within the scene. We demonstrate that vision achieves this goal via a specialized perceptual system for object segmentation that is one and the same with the feature extraction system: immediately after information is relayed to cortex by the eyes, the process of reconstructing image content from local features is controlled by a dedicated inferential mechanism that attempts to recover the underlying environmental structure; perception is quickly organized around the operation of this mechanism, which becomes the primary contextual influence on image reconstruction. The integrated nature of this perceptual mechanism defies current notions of separate top-down and bottom-up processes, offering a fresh view of how human vision operates on natural signals.

## Introduction

**Fig 1:**
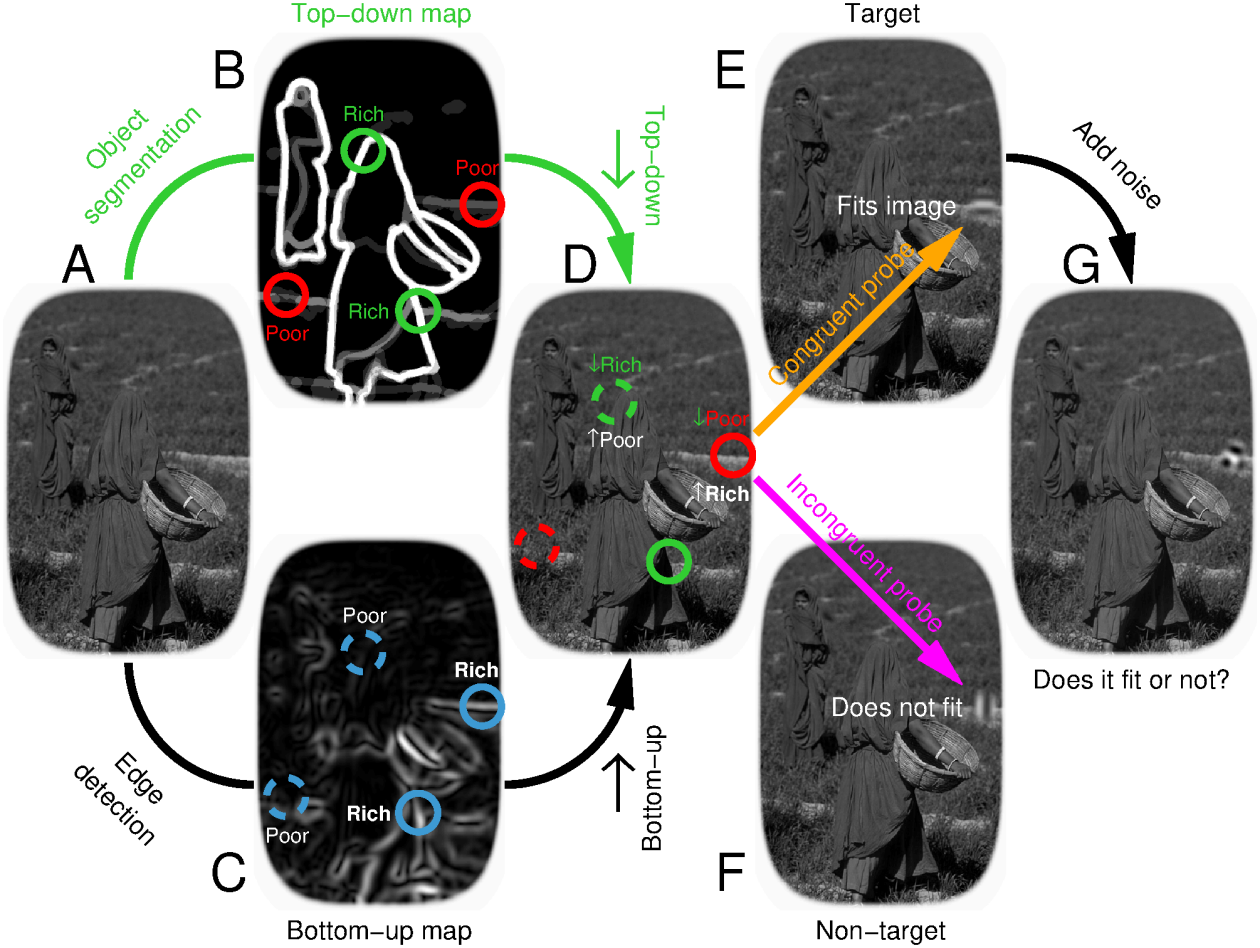
Mapping features from natural scenes. Intensity (brightness) on the top-down map (**B**) reflects saliency of perceptual object representation within the original scene [5, 6] (**A**), while the bottom-up map (**C**) indicates edge energy content [3, 7]. We identify 4 locations that are rich/poor on the top-down map (green/red circles in **B**) and/or rich/poor on the bottom-up map (solid/dashed circles in **C**); the two locations indicated by dashed-green and solid-red circles in **D** are rich on one map and poor on the other. An oriented wavelet is inserted at one location in congruent (**E**) or incongruent (**F**) configuration, orientation noise is added [8] (**G**), and observers must determine whether probe is congruent or not [9, 10].

Consider the image in Fig 1A. During the twentieth century, knowledge of how it may be represented in early visual cortex was galvanized by the discovery that neurons respond to specific features defining the image, such as the orientation and size of its edges and lines [1]. In its simplest form [2], this representation may resemble the feature map in Fig 1C, where the intensity of each location mimics the response of a human edge detector positioned within that region of the image [3, 4].

Our current understanding of cortical feature encoding is much richer than Fig 1C, extending to gain control [11], surround modulation [12], attentional effects [13], crowding [14], and many other phenomena [4, 15]. Nevertheless, whether these additional factors are included or left out, there remains a fundamental problem with feature-driven representations such as Fig 1C: they fail to capture the essential structure of the underlying scene in its relevance to perception and behaviour [16]. For the purpose of relating to this image in the form of scene understanding and potential motor interaction [17–19], our representation of its content is better captured by the map in Fig 1B: the critical boundaries are those that define the 2 human characters, while everything else is of incidental significance [16, 20, 21].

Where and how, specifically, does Fig 1C fail in capturing Fig 1B? Consider the 4 locations indicated by circles in **C**. The 2 solid circles correspond to well-defined boundaries in the physical stimulus: large luminance transitions occur at those locations in the original image (**A**) so that they are richly represented in the edge map of **C**. On the contrary, the 2 dashed circles correspond to boundaries that barely exist within the physical stimulus: locally within the original image, there is little to indicate that a boundary is present at those locations. This is not to say, however, that those boundaries do not exist at a different level of representation: they do exist, but at the level of the scene representation afforded by our mind [20], as indicated by the top green circle in Fig 1B. That specific location marks the boundary between the person in the foreground and the landscape in the background, which is a critical demarcation for representing scene content and supporting image interpretation [22, 23]. It is, however, nearly invisible within the edge map in **C** (top dashed circle). A complementary inconsistency between Fig 1B and 1C is indicated by the red circle on the right-hand side of **B**: this location corresponds to an irrelevant boundary for scene understanding (i.e., poorly represented in Fig 1B), yet it is well-defined in the physical image (richly represented within the edge map in **C**, as indicated by the solid circle on the right-hand side of that image).

How are the two representational levels cartooned in Fig 1B and 1C combined in the visual system? Recent electrophysiological measurements from visual cortex have established that neuronal response properties are sensitive to natural signals [18, 24], thus consolidating the notion that cortical neurons must be viewed as adaptive devices under the control of both bottom-up and top-down information [25, 26]. But how do these flexible cortical effects impact human behaviour? In other words, what are the perceptual signatures of the neuronal effects associated with natural stimulation? We know surprisingly little about this fundamental question [27]. To make progress in this direction, here we deliberately focus on a simplest visual task: reconstructing the local orientation content of a corrupted image region (Fig 1E–1G). This choice of perceptual operation enables us to investigate early visual mechanisms using established low-level tools, while at the same time recasting the ensuing characterization into the higher-level coordinates defined by the natural scene [10], so as to gauge the interplay between the 2 representational levels.

Surprisingly, we find that human reconstruction of local image regions is almost exclusively controlled by the kind of scene representation exemplified by Fig 1B, with only limited signatures of the low-level account returned by Fig 1C. The control exerted by the object-based segmentation map of Fig 1B operates very quickly (within the first 100 ms) and is not altered by spatial attention. In combination with electrophysiological recordings of scalp signals, these results offer a new perspective on the notion of how so-called bottom-up and top-down representations may interface in human vision [20, 28]. They suggest that the conceptual compartmentalization associated with the bottom-up/top-down dichotomy may be more productively replaced by regarding these two processing modes as intimately integrated into a single adaptive mechanism [25, 26, 29], possibly providing a more appropriate framework for understanding early visual perception in humans.

## Methods

### Observers and data mass

All experiments have been approved by the CERB committee (ID470) at the University of Aberdeen and were conducted according to the principles expressed in the Declaration of Helsinki. We tested 8 naive observers (3 males); of these, 6 took part in the double-pass experiments and 7 took part in the electroencephalogram (EEG) experiments. We attempted to bring electrodes into contact with the scalp of the remaining observer; however, this was not possible due to thick hair growth (dreadlocks) that caused high electrode noise and unreliable contact; this observer was therefore excluded from subsequent EEG experiments immediately after the first (unsuccessful) attempt. We collected a total of approximately 310,000 trials, of which approximately 30,000 (>4,000 per observer) during the EEG experiments (evenly divided between intact and cut-out scenes) and approximately 21,000 (>3,500 per observer) during double-pass experiments. For the psychophysical experiments, this corresponds to approximately 50 hours of data collection per observer. For the EEG experiments, 6 observers completed 4 sessions lasting 3 hours each, while the remaining observer completed 2 sessions. Observers were paid 9 EUR/hour for data collection in the psychophysical experiments and 20 EUR/hour for participation in the EEG experiments.

### Presentation protocol and task

All scenes were rescaled to have the same contrast energy; when projected onto the CRT monitor (Iiyama Vision Master Pro 500) by ViSaGe hardware (Cambridge Research Systems), they spanned a luminance range of 4–60 cd/m^2^ against a gray background of 32 cd/m^2^ and occupied (width × height) 13° × 20° or 20° × 13° (depending on whether the scene was in portrait or landscape format) at the adopted viewing distance of 57 cm. Stimulus duration was 300 ms except for a subset of the experiments (approximately 30% of total data mass) during which it was deliberately reduced on a near-logarithmic scale (200, 100, 50, 30, 20, 10 ms) to study the impact of this parameter. Before being displayed, the stimulus could be flipped around its vertical axis (left-right with respect to fixation) with 50% probability (randomly and independently selected on every trial), so that each probe location/type could appear within either left or right hemifield with equal probability. On each trial, observers saw one natural scene containing either congruent (Fig 1E) or incongruent probe (Fig 1F; see below for details on probe design). The scene was centered on a fixation cross that never disappeared. Observers were required to press one of two buttons to indicate whether the probe was congruent or incongruent (this task was worded to them as “determine whether the orientation of the texture within the probe is aligned with the scene, or is orthogonal to the scene”). Their response was followed by trial-by-trial feedback (correct/incorrect) and the next trial was initiated after a random delay uniformly distributed between 200 and 400 ms. Feedback was introduced to push observers into their optimal performance regime so that interpretation of sensitivity (d′) measurements would not be confounded by extraneous factors such as lack of motivation and/or response bias [30] (refer to S1 Text and S3 Fig for detailed analysis of response bias effects). At the end of each block (100 trials), observers were provided with a summary of their overall performance (% of correct responses on the last block as well as across all blocks).

### Primary stimulus design

#### Construction of bottom-up/top-down maps

Our goal was to associate each natural image with 2 maps: the “bottom-up” map, detailing the degree of low-level edge definition at each location within the physical image; the “top-down” map, reflecting the perceptual significance of each location within the image for segmenting and reconstructing the layout of the scene. We express size as [x,y] where x is in degrees of visual angle and y is in percentage units of the smaller side of the natural scene (measuring 13°); we include the latter specification to ease interpretation of how different stimulus elements relate to each other. Images were obtained from the Berkeley Segmentation Dataset [5] (BSD500), a database that is available in the public domain and downloadable from https://www2.eecs.berkeley.edu/Research/Projects/CS/vision/bsds/. Images were then processed by purpose-written software (Matlab) for determining adequate probe insertion points. Except for designing the algorithm and selecting appropriate initialization parameters, there was no human intervention, and the algorithm was fully automated. All scenes were converted to gray-level images. The algorithm built 2 maps from each scene. The bottom-up map was constructed by processing the image with a Sobel-like differentiation filter [7] measuring (active area) ∼ [0.9°,7%]; an example is shown in Fig 1C where filter output (normalized to range between 0 and 1) scales with surface intensity (increasing from dark to bright). The top-down map was constructed by combining segmentations from different BSD500 participants. Lines within individual segmentations were first thickened via blurring/thresholding to measure ∼ [0.5°,4%] (line width) and subsequently combined across participants by assigning to each pixel the proportion of participants for whom that pixel corresponded to a line. An example is shown in Fig 1B where pixel value (ranging between 0 and 1) scales with surface intensity: darkest value (0) for pixels not marked by any participant, brightest value (1) for pixels marked by all participants.

#### Identification of target insertion points

The algorithm for selecting insertion points was designed to identify 4 points per image, one for each combination of rich versus poor on top-down versus bottom-up maps: an insertion point corresponding to a poor location on the top-down map and a rich location on the bottom-up map, which we refer to as poor/rich for top-down/bottom-up content; 3 more points corresponding to poor/poor, rich/poor, and rich/rich. For each nonzero pixel on the top-down map, we computed the elongation of nonzero pixels within a square region (sized [1.6°,12%]) centred on that pixel. Elongation ranges between 0 (circle-like) and 1 (line-like); it specifies the half-focal separation of the ellipse that takes the same second-moments as the analyzed region, which we refer to as the “best-fitting” ellipse. We then excluded all pixels with elongation <0.9 and intensity <0.1 on the bottom-up map (i.e., those that did not conform to an elongated boundary), as well as those located near the edge of the image (within **∼** [2°,15%] from edge). The remaining pixels were labelled top-down “rich” if their value on the top-down map was 1, or “poor” if it was <1. We then selected, among top-down rich pixels, the pixel corresponding to the smallest value on the bottom-up map. We call the latter value *v*. We also selected, among top-down poor pixels, the pixel corresponding to the value on the bottom-up map that was closest to *v*. These two selections were labelled respectively rich/poor and poor/poor for top-down/bottom-up content. We then selected, among poor pixels on the top-down map, the pixel corresponding to the largest value on the bottom-up map. We call the latter value *V*. We also selected, among rich pixels on the top-down map, the pixel corresponding to the value on the bottom-up map that was closest to *V*. These two selections were labelled respectively poor/rich and rich/rich for top-down/bottom-up content. We further imposed the constraint that the 4 selected insertion points should not be within [1.6°,12%] of each other to reduce potential overlap of the image elements targeted by the different insertions. Once an insertion point is selected, we identified the orientation of the best-fitting ellipse to non-zero pixels within a square [0.8°,6%] region centred on that point, and labelled it as the local congruent orientation. Of the 500 images within BSD500, 53 did not contain enough pixels with characteristics that satisfied the above constraints, and were therefore excluded.

#### Probe design and insertion

Our goal was to generate a low-level stimulus that could be smoothly grafted into the natural scene, enabling us to retain full control over the statistical properties of the probe while at the same time embedding it within a complex stimulus (i.e. the natural scene) for which we lack the same degree of control. To achieve this goal, we build upon prior work with both isolated [8] and embedded [9, 10] low-level elements. Probes (see example in Fig 1G) consisted of 16 superimposed pseudo-Gabor wavelets at 16 different orientations uniformly spanning the 0-*π* range, each taking a random phase. Carrier spatial frequency was fixed at ∼1 cycle/degree. The envelope was constant over a circular region spanning ∼ [1.6°,12%] (diameter); outside this region, it decreased smoothly to 0 following a Gaussian envelope of SD ∼ [0.1°,0.8%]. The 16 contrast values assigned to the different wavelets on each trial are denoted by vector **s**^**[*q*]**^ (*q* = 1 for congruent probe, *q* = 0 for incongruent probe; see next section for more details). With relation to this vector representation, the congruent orientation corresponds to the fifth entry into the vector; in actual image space, the congruent orientation is selected as the best match to the orientation associated with the insertion point (see above), while the incongruent orientation is always orthogonal to the congruent orientation. The probe was smoothly inserted (by using wavelet envelope to control probe/image ratio contribution to image) into the local region of the natural scene identified by the automated procedure detailed above (see examples in Fig 1E and 1F for congruent and incongruent probes respectively). Probe insertion density gradually declined away from fixation (see S2A and S2B Fig), partly as a result of stimulus geometry.

#### Injection of orientation noise

Our goal was to perturb the orientation content of the probe via random fluctuations of different orientation components, for the purpose of subsequently establishing the link between specific fluctuations in the stimulus on the one hand, and the associated response choices made by human observers on the other hand (psychophysical reverse correlation [31]). On each trial **s**^**[*q*]**^ = **t**^**[*q*]**^ + **n**^**[*q*]**^: the contrast distribution across orientation consisted of a target signal **t** (deterministic) summed onto a noise sample **n** (statistically defined and therefore different on every trial). The target signal vector **t**^**[*q*]**^ consisted of 0's everywhere except the fifth entry when *q* = 1 (congruent probe) or the thirteenth entry when *q* = 0 (incongruent probe), which was assigned a value denoted by *ρ* (target intensity). Each entry of the noise vector **n** followed a Gaussian distribution with mean 3% (contrast units) and SD 0.7% clipped to ± 4 SD. We adjusted *ρ* individually for each subject to target threshold performance; when expressed as multiple of noise mean, *ρ* was approximately 4 (mean across observers; equivalent to approximately 12% contrast). When the task was particularly challenging due to specific manipulations (e.g., extremely short durations), noise was removed (**n** = 0) and *ρ* ∼ 18% (contrast units); this large SNR (effectively ∞) was applied on approximately 20% of total data mass to ensure that overall performance was above chance (d′>0).

### Spatial cueing

We designed a cueing paradigm to manipulate spatial attention so that observers were given the opportunity to deploy attention to the local probe on some trials but not others (each trial being of other type with equal probability). On “precue” trials, the main stimulus described above was preceded by a spatial cue (duration 100 ms) consisting of a blob (defined by probe envelope and therefore matched to probe size) that colocalized with the probe (see S1 Video); the interval between cue and main stimulus was uniformly distributed between 150 and 300 ms. On “postcue” trials, the same cue was presented but it followed the main stimulus (after an interval specified using the same parameters adopted for precue trials). Under particularly challenging task conditions (large gaps, short durations, power-only stimuli), we only adopted the precue condition to help performance maintain above-chance levels. Spatial cueing was not adopted with zooming stimuli (see below for detailed description) to avoid introducing additional dynamic elements to an already dynamic stimulus; cueing was redundant in this case because the zooming process implicitly cues probe location.

### Scene manipulations

The main effects reported in this study are measured with scenes that, except for probe insertion, retain their natural characteristics. It is important to determine exactly which of those many characteristics play a role in driving the top-down effect [32]. To achieve this goal, we manipulated the global appearance of the scene.

#### Lowpass/highpass filtering

Lowpass filtering attenuated power uniformly for all frequencies above 0.5 cycles/deg by approximately 70%; highpass filtering reduced power progressively for all frequencies below 7 cycles/deg, starting at approximately 10% attenuation for 7 cycles/deg and progressing to 100% attenuation for frequencies below 0.5 cycles/deg (those preserved by the lowpass filter). A circular region of size ∼ [3.1°,24%] (diameter of tapered envelope) immediately surrounding the probe was left intact.

#### Warping

Warping was applied by first selecting 40 lattice points uniformly spanning the scene with the exclusion of those within [3.2°,25%] of the probe insertion point. The latter exclusion was adopted to ensure that the warped image would merge smoothly with the circular region immediately surrounding the probe, which was left intact as detailed above for filtered images. The remaining points served as centers for 2 image warping manipulations: swirling and lensing. Swirling consists of local rotation controlled by an angle that depends on distance from center. Lensing consists of an exponential distortion of local coordinates not dissimilar from converting linear to log coordinates to emphasize values near the origin. Each selected center could either be swirled or lensed, the distortion chosen randomly and independently for each center, but only performed once for a given scene and insertion point (i.e., the warped scene did not change from trial to trial). The degree of warping was controlled by allowing distortions to extend over a limited region surrounding each center of application; the region was defined by a Gaussian envelope with standard deviation [0.6°,5%] for weak warping and [1.2°,10%] for strong warping.

#### Cut-out/Lines

For the “lines” manipulation, the top-down map was thresholded (>0) to binary, i.e., all boundaries were assigned the largest luminance value (60 cd/m^2^) if they had been selected by at least 1 participant in BSD500. For the “cut-out” manipulation, each region defined by those boundaries was assigned a constant luminance value chosen from a predetermined set of values uniformly spanning the entire luminance range (4–60 cd/m^2^) and randomly permuted. This means that 1) no two regions took the same luminance value, ensuring that different regions would never merge; and 2) the luminance difference between any two regions was above visibility threshold. Similarly to warping, randomization was only applied once for any given natural scene, and the same cut-out scene was then used on multiple trials. For these 2 manipulations, the region surrounding the probe was not preserved. The latter detail is important particularly in relation to the cut-out manipulation, because it ensures that all potential low-level cues in the original scene (including second-order ones) had been eliminated (see S1 Text).

#### Phase/power scrambling

For each scene in the database, we generated an image consisting of white noise (each pixel being independently assigned a random luminance value). We then replaced the phase spectrum of the scene with that from the white-noise image to generate power-only scenes, or we replaced the power spectrum to generate phase-only scenes. Similarly to filtered scenes, the region surrounding the probe was preserved.

### Zooming stimuli

To study the spatiotemporal characteristics of scene-probe interactions, we designed “zooming” variants of the stimulus where a smooth transition is enacted between a probeless scene and a sceneless probe. We generated a zooming envelope for each probe insertion point via thin-plate spline warping [33] of reference points specified around scene edge and probe edge. This envelope was constructed so that it would toggle between 2 views: one in which most of the scene was visible but the probe was not visible, and one in which only the probe region was visible. All edges of visible regions were tapered. In the zoom-in variant, the envelope smoothly transitioned from the scene-without-probe image to the probe-without-scene image over 30 frames (total duration 300 ms) or 10 frames (100 ms) in the “long” and “short” configurations, respectively. The opposite direction was applied for the zoom-out variant. Please refer to S2 Video for examples of both zoom-in/zoom-out and long/short stimuli.

### Gap stimuli and short stimulus presentations

The zooming stimulus compounds spatial with temporal manipulations. We studied these 2 factors separately by either introducing a gap between probe and scene (spatial manipulation) or decreasing stimulus duration (temporal manipulation). For spatial gaps, the probe was surrounded by a circular mean-luminance region smoothly merging into the scene and extending out to a diameter of (in units of probe diameter size) 1.25, 1.5, 2.5, and 5. The discrimination task was particularly challenging for larger gap sizes (covering up to approximately 20% of the area occupied by the scene), requiring that noise be removed from the probe in order to support above-chance performance. Some observers were, nevertheless, unable to perform above chance under those conditions; those instances were excluded from the individual-observer analysis by removing all log-ratios associated with d′ ≤ 0. A similar issue arose in connection with very brief stimuli (10–20 ms); again, those instances were excluded from the composite analysis. We nevertheless display estimates for all observers because, for a given set of conditions across which log-ratios were computed (e.g., 10 ms and 20 ms), all observers returned a viable estimate for at least one of the conditions within that set (e.g., 10 ms or 20 ms).

### Orientation tuning

Above-chance performance in the congruent/incongruent task requires observers to assign perceptual weight to different orientation channels in a nonuniform fashion; the weight profile can be summarized in the form of an orientation tuning function. To derive an empirical estimate of tuning characteristics, we capitalized on the presence of orientation noise within the probe combined with the perceptual coupling between specific noise perturbations and the trial-by-trial response generated by the human observer [8, 31, 34].

#### Derivation of tuning functions

To maximize data mass, we pooled noise samples from all configurations that demonstrated top-down effects: intact, inverted (upside-down), cut-out, highpass, phase-only, intact/cut-out during EEG experiments (total of approximately 140,000 trials). Each noise sample is denoted by **n**^[*q*,*z*]^: the sample added to congruent (*q* = 1) or incongruent (*q* = 0) probe on a trial to which the observer responded correctly (*z* = 1) or incorrectly (*z* = 0). The corresponding orientation tuning function **p** is derived via the standard formula for combining averages from stimulus-response classes [34]:

$$p=\langle n^{\left[1,1\right]}\rangle +\langle n^{\left[0,0\right]}\rangle -\langle n^{\left[1,0\right]}\rangle -\langle n^{\left[0,1\right]}\rangle$$

where 〈〉 is average across trials of the indexed type. Under some commonly adopted assumptions regarding the nature of sensory transduction [31, 34], **p** provides a description of the “perceptual weight” assigned by human observers to different parts of the stimulus. For example, a peak corresponding to the fifth entry of vector **p** indicates that observers were more likely to report the probe as being “congruent” when noise orientation energy happened (by chance) to be more prominent within the orientation range corresponding to the congruent orientation (fifth entry into vector **n**). To increase measurement SNR, the tuning functions have been symmetrically averaged around congruent/incongruent coordinates under the reasonable assumption that observers show no bias either clockwise or counterclockwise of the 2 orientations defining the task. The validity of this assumption (which is a logical necessity-given stimulus/task symmetry) was verified empirically by attempting to detect differences between values immediately clockwise and counterclockwise of the 2 reference orientations (congruent/incongruent); all attempts failed without approaching statistical significance.

#### Retuning index

Under the simplest signal detection theory (SDT) model [35] that is applicable to the problem at hand, the expected sensitivity of the orientation tuning function is controlled by the differential energy output to congruent and incongruent signals, normalized by the overall energy output to signal plus noise: [**p**(13) − **p**(5)]^2^ / Σ**p**^2^ (entries 5 and 13 into vector **p** correspond to congruent/incongruent orientations). This metric is designed to be ≥ 0 (effectively >0) to enable log-ratio computation.

#### Retuning model

Stimulus image (2D) **S** was generated using specifications identical to those adopted in the psychophysical experiments (except for lower SNR) and processed by a quadrature-pair operator:

$${\langle S,W(f_{t},0)\rangle }^{2}+{\langle S,W(f_{t},1)\rangle }^{2}$$

where 〈,〉 is 2D inner product (also termed Frobenius) and filter **W**(*f*_*t*_, *p*) is a Gabor wavelet of spatial frequency *f* oriented along congruent (*t* = 1) or incongruent (*t* = 0) axes at even (*p* = 0) or odd (*p* = 1) phases. We define the output of this operation *r*_*t*_. The decision variable generated by the model is $\frac{r_{1}}{r_{1}+r_{0}}$, i.e., the output from the congruent-oriented operator normalized by the summed output of congruent and incongruent operators; when it exceeds a prespecified threshold value, the model responds “congruent,” otherwise it responds “incongruent.” The threshold value is the average decision variable across 8,000 trials, half with **S** containing a congruent signal and half an incongruent signal (unbiased criterion). In the “Poor” state *f*_1_ = 4 and *f*_0_ = 21 in multiples of the spatial frequency assigned to the target signal carried by **S**; in the “Rich” state *f*_1_ = 1 and *f*_0_ = 3. In words, the model filter aligned with the congruent signal undergoes Poor→Rich sharpening from 4 × broader than the signal (mildly tuned) to match the signal (*f*_1_ = 1), while the model filter aligned with the incongruent signal sharpens from virtually untuned (21 × broader than congruent/incongruent signal) to mildly tuned (3 × broader).

### Internal noise estimation

We performed additional experiments specifically designed to enable internal noise estimates using double-pass protocols [36, 37]. Observers collected 100-trial blocks during which the second 50 trials (51–100) were identical to the first 50 trials (1–50) except for random permutation of their order [38]. The degree to which observers reproduce their own responses to the first pass of 50 trials when those trials are re-presented during the second pass is controlled by their intrinsic variability [39]. Under the standard SDT model [35] where this variability is captured by a late additive internal noise source, the intensity of the latter can be estimated by reverse application of the SDT model to the empirically measured values of percent correct and percent agreement [36] (% of trials on which observers gave the same response to 2 identical passes). Because participants demonstrated an appreciable amount of response bias (see S3 Fig for details), the procedure originally developed for the unbiased case [36] was adapted to the yes–no single-interval protocol used in this study via application of established techniques [34, 40].

### Segmentation algorithms

We applied the following 6 computer vision algorithms from published literature: visual saliency [3] (Itti-Koch), graph-based visual saliency [41] (GBVS), hierarchical segmentation [5] (gPb-HS), normalized cuts [42] (nCuts), contour detection using superpixel-based candidates and hierarchical visual cues [43] (HVC), conditional random fields as recurrent neural networks [23] (CRF-RNN). HVC and gPb-HS had already been applied to BSD500; for those, we obtained results from the algorithm creators. For the remaining models, we implemented them on our hardware and fed them images from BSD500. For some of these algorithms, the output maps are unsuitable for determining whether model output scales with the graded sensitivity measurements via correlation due to the following issues: 1) the output is binary rather than continuous; 2) the identified boundaries are excessively thin (1-pixel width) so that a specific probe insertion point may fail to return a large value on the model map due to slight misregistration, even though the model has effectively labelled that point in the image as belonging to an identified boundary. These issues arose in connection with nCuts, gPb-HS, and CRF-RNN. We thickened the boundaries generated by these models via convolution with an averager of size ∼[0.13°,1%] (gPb-HS), ∼[0.4°,3%] (nCuts), or ∼[0.5°,4%] (CRF-RNN). We verified that the specific choice of thickening parameter/method did not impact the significance of the correlation between model output and human sensitivity. For the purpose of computing rich/poor log-ratios, model output is classified as rich if it exceeds its median value across all probe insertion points, and poor otherwise.

### EEG

#### Stimulus adjustments

Stimulus/task design was very similar to that described above for experiments not involving neuroimaging except for 3 important adjustments geared towards the EEG: 1) because our focus was on probe-specific waveforms obtained via differential contralateral-ipsilateral activity, we replaced the CRT monitor with a wide-field LCD monitor (active area 88 × 50 cm, luminance range 0–200 cd/m^2^) to maximize probe eccentricity via approximately 1.8 × scaling (in degrees) of the visual stimulus (see [44] for advantages/disadvantages associated with using TFT monitors in EEG measurements); 2) we cued probe location via slight red tinting (1:4 tint:image ratio) to avoid asynchronous cue presentation (the latter design, used in the behavioural experiments, would result in overlapping visual evoked potentials (VEPs) from cue and stimulus, reducing interpretability of the EEG waveform [44]); 3) the key press (immediately followed by feedback lasting 100 ms) triggered presentation of the next stimulus after a random interval uniformly distributed between 1.5 and 2 seconds (this longer interval was adopted to avoid ERP overlap across trials).

#### Data acquisition/analysis

EEG was recorded from 32 active electrodes (10/20 layout) at a sampling rate of 256 Hz by a BrainAmp DC-amplifier (Biosemi). Data analysis was performed with Fieldtrip; we confirmed that virtually identical results were returned by Eeglab. We extracted 1-second segments (re-referenced to Cz) from each trial starting at 200 ms before stimulus onset. Baseline was subtracted from the 200-ms interval preceding the stimulus. We applied 2 different (causal [45]) filtering regimes for highpass/lowpass cut-offs: 1/20 Hz and 0.5/40 Hz. To steer our analysis towards effects with clear contralateral/ipsilateral characteristics, we linearly rescaled waveforms from individual trials by the distance of the probe from the vertical meridian before averaging across trials (the greater the distance, the larger the waveform). The effects we report do not depend on this procedure, as demonstrated by the results obtained with artefact rejection for which rescaling was not applied. When artefact rejection was applied, it involved an automatic Fieldtrip routine that excluded EOG, muscle, and jump artefacts with conservative parameters that led to a high trial rejection rate of approximately 19%. We confirmed that more lax parameter choices (resulting in lower rejection rates) produced equivalent results. We do not detail this procedure further because artefact rejection made no difference to the primary results. After trial averaging, we obtained 4 waveforms (1 for each probe insertion type) from each electrode in each observer and normalized the traces separately to have equal RMS. Further data analysis involved simple waveform subtractions and/or pooling as described in the main text. Electrodes Oz/Fz are not included because our focus is on lateralized activity [46].

#### Confirmatory experiment with cut-out images

Except for replacing natural scenes with their cut-out variants to enhance the conceptual significance of the results, this experiment was deliberately conducted in such a manner as to match the original experiment as closely as possible. The same observers who participated in the original experiment were asked to attend the same number of 3-hour sessions in order to match data mass, resulting in almost identical number of trials for the 2 conditions (14,300 versus 15,700 for natural and cut-out scenes, respectively). All details of the experimental protocol were matched (within margin of inevitable differences such as exact electrode placement, quality of electrophysiological signal, time of day, and similar factors), and identical analysis tools were applied to obtain the processed results.

### Statistical analysis

We adopt a combination of confidence intervals and *p*-values returned by two-tailed Wilcoxon signed-rank tests to avoid the limitations associated with *p*-values alone [47, 48]. There are only a few instances in this study where these two approaches are in conflict; we highlight those instances explicitly and investigate them further. The experiments were designed so that the null hypothesis adopted for the Wilcoxon tests would be transparently and unambiguously defined as involving no difference between 2 measurements of the same variable under 2 different conditions. In general, the primary effect reported in this study (top-down modulation of sensitivity) is sufficiently large and robust to eliminate any concern as to its statistical reliability. To verify robustness/replicability, we also adopt a confirmatory approach where we tackle the primary result from multiple directions.

## Results

### Image reconstruction is controlled by top-down information

We grafted an oriented wavelet into a natural scene (Fig 1E and 1F), and asked human observers to determine whether its orientation was congruent (**E**) or incongruent (**F**) with the structure locally defined by the scene [10] (see Methods). The orientation content of the wavelet was disrupted via the addition of orientation noise [8, 10] (Fig 1G). This manipulation engaged observers in active image reconstruction of a locally corrupted signal and supported noise-based characterization of relevant phenomena using state-of-the-art psychophysical tools [8, 31].

The grafted wavelet, which we refer to as the “probe” stimulus, could be inserted at 1 of 4 different locations within each image from a database of approximately 450 scenes. The 4 insertion points represented all combinations of poor versus rich locations within 2 maps derived from the natural scene, which we refer to as the “bottom-up” and “top-down” maps, and were selected across the image database so that map values could be independently manipulated (S1 Fig). The “bottom-up” map (Fig 1C) reflects energy content as returned by common edge detection algorithms [7] (see Methods); designated poor/rich locations on this map are indicated by dashed/solid circles. The “top-down” map (Fig 1B) reflects consensus across human participants when asked to segment individual scenes by marking relevant boundaries as part of the Berkeley Segmentation Dataset project [5] (BSD500). Rich regions (indicated by green circles) denote boundaries marked by all participants, while poor regions (red circles) were selected by some participants but not others (see Methods). We rely on the “top-down” map as a proxy for the segmented representation of the scene afforded by the human visual system [5, 6]. Our choice of the terms “bottom-up” and “top-down” was motivated by lack of better options [30, 49], rather than accurate descriptive purposes. As we discuss later in the article, we do not think these terms are adequate for describing the role played by the information contained within the maps, but they are nevertheless useful as labels for facilitating exposition of the results.

**Fig 2:**
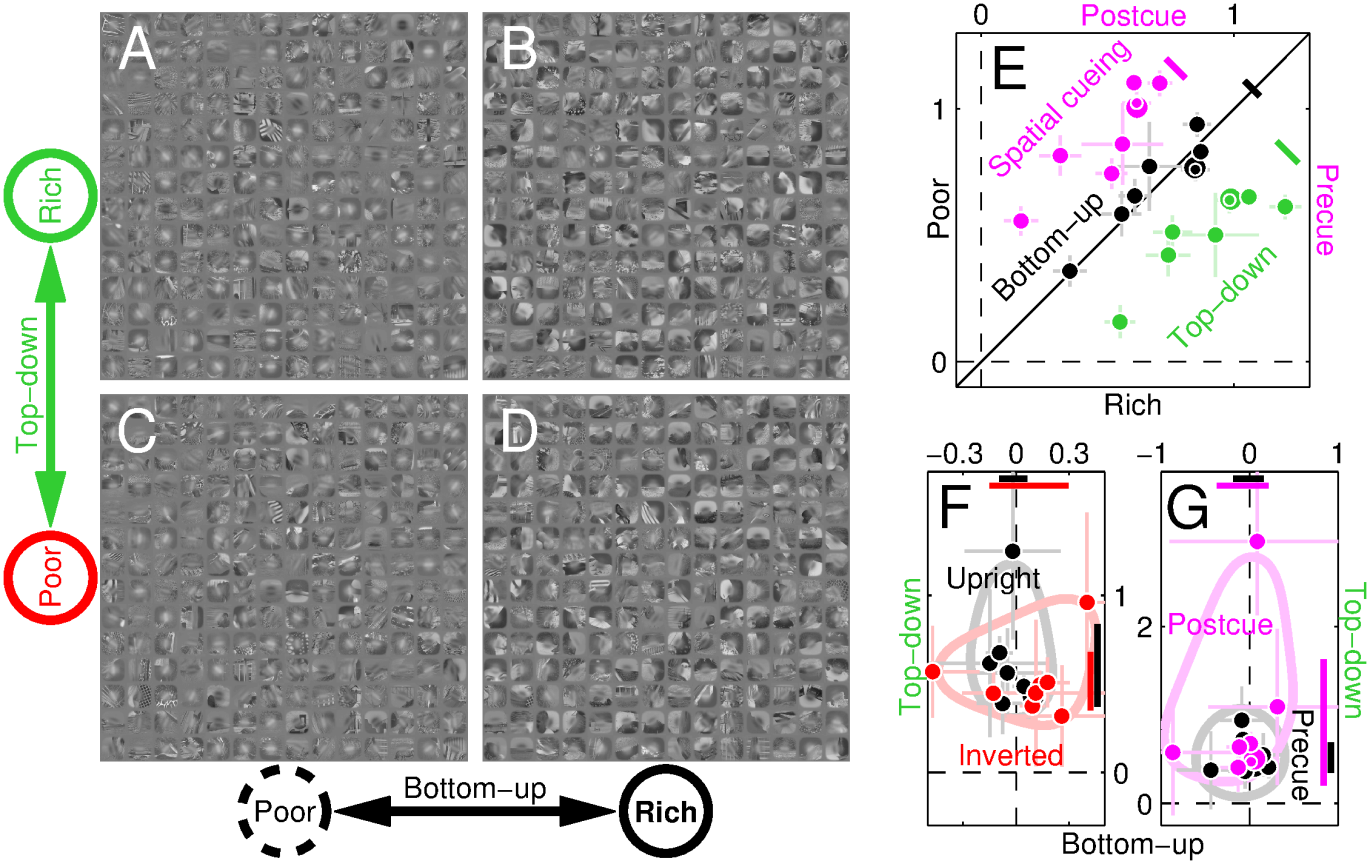
Performance is driven by top-down map. **A**-**D** show collections of image regions (approximately 3 × probe size) surrounding probe insertion points (with embedded congruent probe) at rich/poor locations on top-down map (**A**-**B** versus **C**-**D**) or bottom-up map (**B**,**D** versus **A**,**C**). The poor→rich transition is perceptually evident across the bottom-up map (left → right). **E** plots sensitivity (d′) for poor (*y* axis) versus rich (*x* axis) locations on the bottom-up (black) or top-down map (green) in individual observers (1 symbol per observer), as well as precue (*y* axis) versus postcue (*x* axis) configurations (magenta). **F**-**G** plot sensitivity rich/poor log-ratios for top-down (*y* axis) and bottom-up (*x* axis) comparisons when scenes were upright or inverted (black or red in **F**) and precued or postcued (black or magenta in **G**). Error bars plot ±1 SEM. Coloured diagonal segments in **E** plot 95% confidence intervals for data projected along negative diagonal. Horizontal/vertical segments near *x*/*y* axes in **F**-**G** plot confidence intervals for bottom-up/top-down log-ratio effects; light-coloured contours indicate data spread for visualization aid. Data for this figure is available from S2 Data.

Fig 2A–2D show small square regions around various probe insertion points from randomly selected scenes. From simply looking at these images, it is apparent that the examples in Fig 2A and 2C, corresponding to poor locations on the bottom-up map, contain visibly less edge-contrast than those in Fig 2B and 2D, corresponding to rich locations on the bottom-up map. This is not surprising: it is the criterion by which insertion points were selected in the first place with reference to the bottom-up map (see Methods). It is more interesting to compare, by the same token of summary visual inspection, the collection in Fig 2A and 2B, corresponding to rich locations on the top-down map, versus that in Fig 2C and 2D, corresponding to poor locations on the top-down map: there is no obvious difference between the 2 collections at this level of inspection. In other words, if we consider the most immediate and perceptually obvious content of the image around the probe, the difference is much greater as we move along the bottom-up map (left to right in Fig 2A–2D) than it is as we move along the top-down map (top to bottom). In fact, there seems to be virtually no change of statistical properties for the latter transition.

It therefore comes as a surprising finding that, when we measure how well human observers are able to reconstruct the orientation of wavelet signals inserted at those locations, their performance displays the opposite trend: there is no difference in sensitivity for rich (*x* axis in Fig 2E) versus poor (*y* axis) locations on the bottom-up map (black symbols scatter around diagonal equality line at *p* = 0.74), while there is a marked difference for the poor–rich comparison on the top-down map (green symbols fall below equality line at *p* < 0.01; see also confidence intervals indicated by black/green diagonal segments). This effect is plotted more compactly in Fig 2F (black symbols) in the form of rich/poor log-ratio values: for the bottom-up comparison (*x* axis), log-ratios scatter around 0 (corresponding to no difference between rich and poor values), while for the top-down comparison (*y* axis) they all fall above 0 (rich > poor; *p* < 0.01; see confidence interval indicated by black segment near *y* axis). It appears that the ability of the human visual system to extract local orientation signals depends greatly on whether those signals correspond to richly versus poorly represented boundaries within the top-down map, and not at all on whether the boundaries are rich or poor on the bottom-up map (see also [50, 51]), even though visual inspection of those local boundaries demonstrates no difference for the former comparison (Fig 2A and 2B versus Fig 2C and 2D) and an easily perceptible difference for the latter (Fig 2B and 2D versus Fig 2A and 2C).

### Top-down effects are reduced when scene intelligibility is degraded

**Fig 3:**
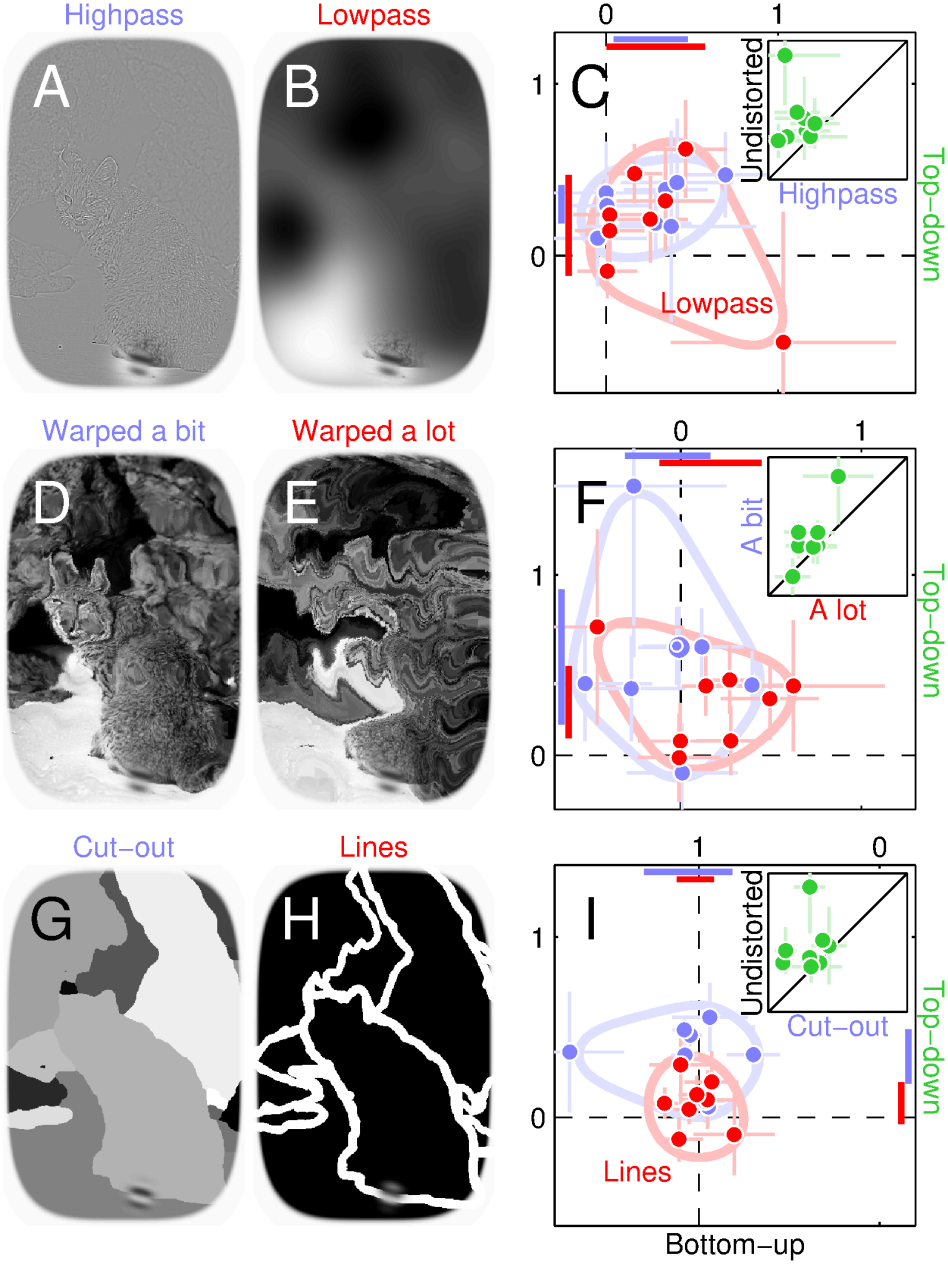
Scene manipulations may eliminate top-down effects and/or produce bottom-up effects. Natural scenes were highpass/lowpass filtered (**A**-**B**), warped a bit or a lot (**D**-**E**), and converted to cut-out or line versions (**G**-**H**). **C**,**F**,**I** are plotted to the conventions adopted in Fig 2F and 2G; insets plot top-down effects for specific comparisons. Data for this figure is available from S2 Data.

We carried out an extensive series of additional experiments to determine whether the top-down effect is robust and how far it generalizes across manipulations of cognitive factors and scene characteristics. We found that it does not require semantic labelling of scene content (it is unaffected by image inversion [52, 53] or contrast reversal [54], see red symbols in Fig 2F and S1 Text), operates independently of spatial attention (it is unaffected by spatial cueing, see Fig 2G, S1 Text and S1 Video) but does depend on specific manipulations of various image properties such as spatial frequency (it is retained with highpass but lost with lowpass scenes, see blue/red symbols/confidence intervals in Fig 3C), orientation (it is reduced by image warping, see Fig 3D–3F), object-boundary definition (it is retained with cut-out scenes but lost when object boundaries are defined solely by lines, see blue/red symbols/confidence intervals in Fig 3I) and others (see S1 Text). For example, although the top-down effect is retained with highpass-filtered images, its magnitude is smaller than observed with undistorted scenes (green symbols in inset to Fig 3C fall above diagonal equality line at *p* < 0.01). Similarly, although this effect is measurable for cut-out scenes, its magnitude is again smaller than observed with intact images (green symbols in inset to Fig 3I fall above equality line at *p* < 0.03).

Interestingly, under some conditions (undistorted scenes) we only observe a top-down effect (Fig 2F), under other conditions top-down and bottom-up effects coexist (highpass scenes), and finally there are conditions for which only the bottom-up effect is observed (lowpass scenes; please refer to S1 Text for detailed statistics including outlier detection). The top-down effect is therefore not a trivial inevitable consequence of task/probe design, and our measurement protocols are adequate for exposing both top-down and bottom-up effects: when one is not observed, this reflects a genuine lack of contribution from the corresponding information source, rather than failure on the part of our methods to resolve its presence.

**Fig 4:**
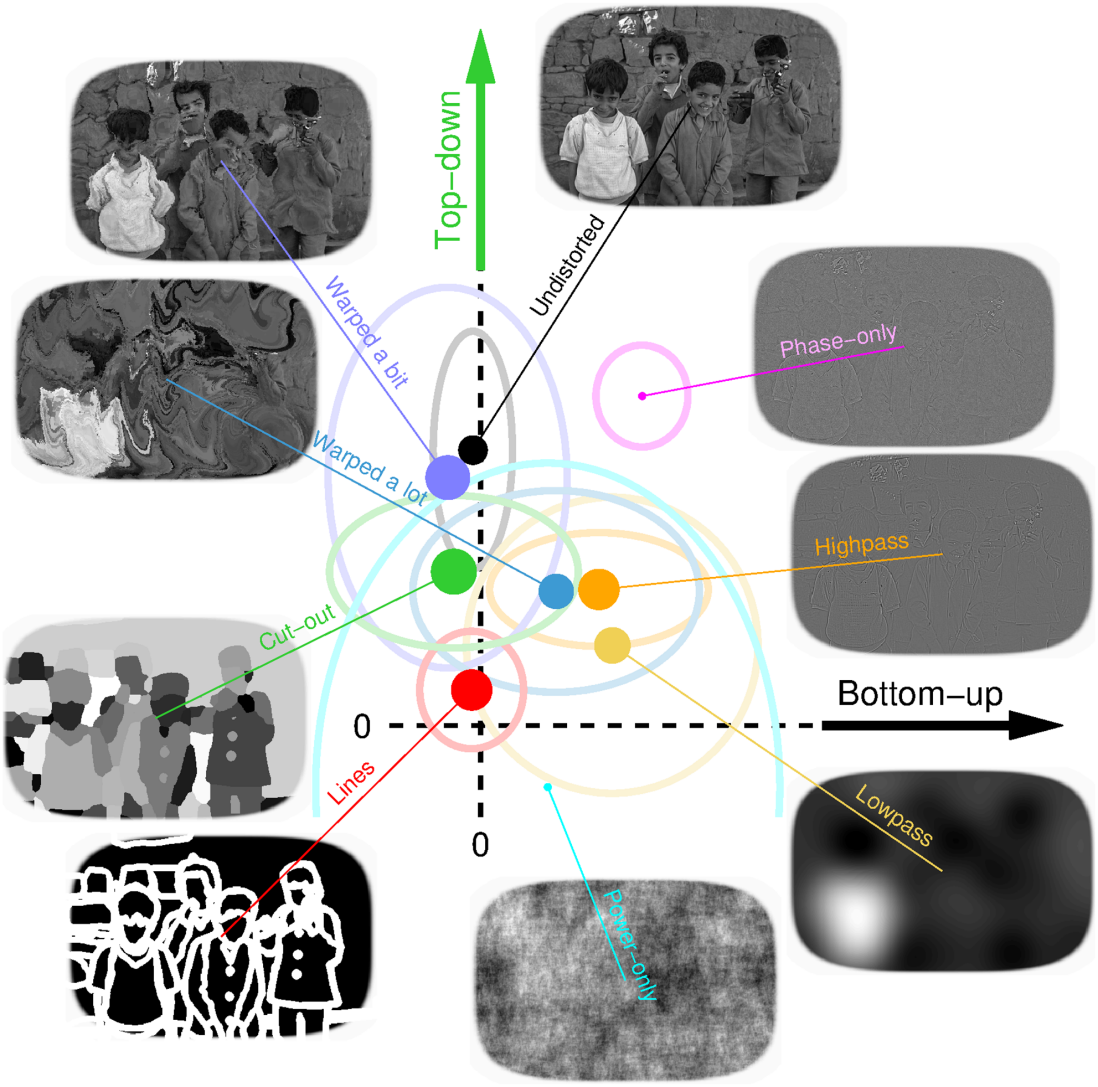
Summary of image manipulations. Top-down (*y* axis) and bottom-up (*x* axis) effects are plotted for all scene manipulations averaged across observers (each symbol shows average for the indicated configuration, ovals plot ±1 SD across observers). Symbol size scales with absolute efficiency [35] (directly proportional to d′ and inversely proportional to stimulus SNR). Data for this figure is available from S2 Data.

We summarize the results of all image manipulations in Fig 4: whenever the natural scene is manipulated in some way, whether by warping its orientation content (purple/blue), filtering its spatial frequency structure (yellow/orange), or artifically perturbing its segmentation content (green/red), the top-down effect is consistently reduced (data points shift downward towards origin), to the extent that it may be entirely eliminated (red/cyan/yellow). This is not to say that the full natural appearance of the image is necessary to observe top-down effects: cut-out scenes, although still interpretable as containing natural elements, do not “look” natural. In general, however, top-down effects were reduced for a number of image manipulations, indicating that their origin involves various aspects of natural scene content. Fig 4 also illustrates that top-down effects were not related to the absolute difficulty of the discrimination task (efficiency is indicated by symbol size; there does not appear to be any lawful relationship between symbol size and symbol position within the graph).

### Top-down effects are ultrafast and spatially distributed

**Fig 5:**
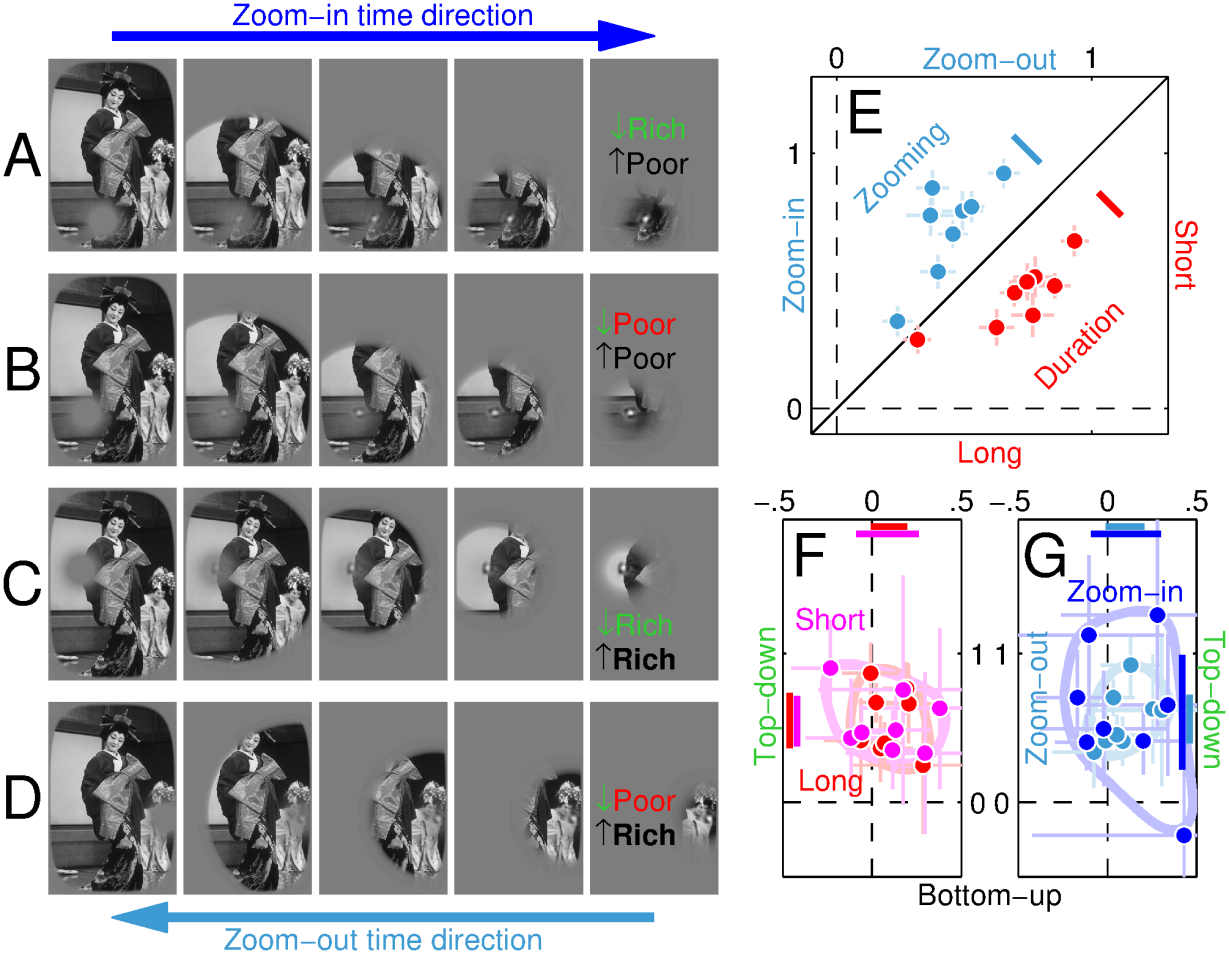
Scene-probe dynamics impacts absolute sensitivity but not differential effects. Zooming stimuli involve smooth transitions from scenes without probes (leftmost icons in **A**-**D**) to probes without scenes (rightmost icons) in either scene-to-probe “zoom-in” direction (left to right in **A**-**D**) or probe-to-scene “zoom-out” direction (right to left). **E** plots sensitivity (d′) for zoom-in (*y* axis) versus zoom-out (*x* axis) configurations (blue symbols) and long-duration (300 ms, *x* axis) versus short-duration (100 ms, *y* axis) stimuli (red) using conventions similar to Fig 2E. **F**-**G** plot corresponding log-ratios using conventions similar to Fig 2F and 2G. Data for this figure is available from S2 Data.

Feedback interpretations of top-down effects [55] may lead to the expectation that these effects should depend on the temporal order of information accrual from the probe in relation to the surrounding natural scene [56]. We tested this prediction by designing “zooming” variants of our stimulus, where a smooth transition was enacted between a probeless scene and a sceneless probe for any probe location and type (Fig 5A–5D; see Methods and S2 Video). We found that, although the spatiotemporal relationship between scene and probe enhances absolute sensitivity in the direction of scene analysis facilitating probe analysis (blue symbols in Fig 5E), the qualitative operation of the system in relation to the top-down/bottom-up differential effect is independent of spatiotemporal dynamics (Fig 5F and 5G).

**Fig 6:**
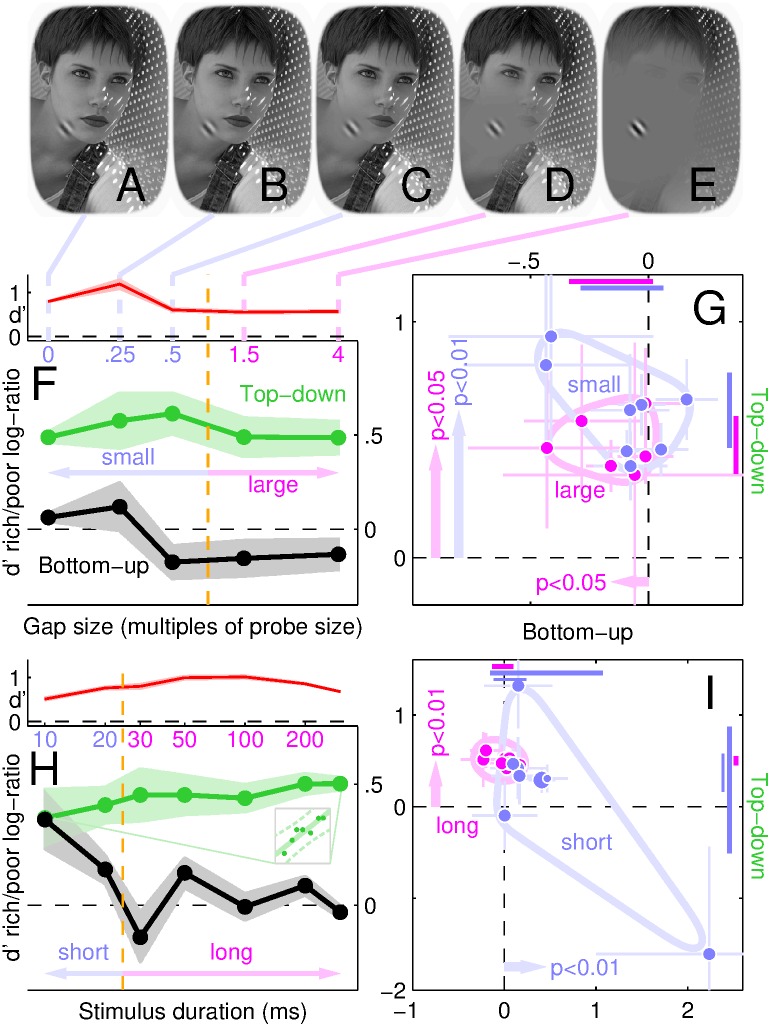
Top-down effect is spatially global (F) but reduced at ultrashort durations (H). **F** plots d′ log-ratios for bottom-up (black) and top-down (green) effects as a function of gap size (*x* axis) for spatial gaps of differing size between probe and scene (**A**-**E**), pooled across observers. Red trace plots overall d′. Shading shows ±1 SEM. **G** plots log-ratios for individual observers (conventions similar to Fig 2F) pooled separately from small (gap < probe, blue) and large (gap > probe, magenta) gap sizes. **H**-**I** show similar measurements for varying stimulus durations, short (< 30 ms, blue) and long (≥30 ms, magenta). Inset to **H** replots green data with rescaled *y* axis to emphasize positive trend (solid line shows best linear fit, dashed lines 95% confidence intervals for fit). Vertical/horizontal arrows in **G**,**I** point to average *y*/*x* values for effects associated with significant *p*-values (<0.05) from Wilcoxon signed-rank test for different than 0 (*p*-values are indicated next to arrow). Thin blue segments near axes in **I** show confidence intervals for blue dataset after removal of data point at bottom-right of panel. Data for this figure is available from S2 Data.

We also investigated spatial and temporal factors independently, rather than compounded in a zooming stimulus. To study space, we inserted a gap between the probe and surrounding scene (Fig 6B–6E); we found no impact on top-down effects up to large gaps (Fig 6F and 6G), indicating that the origin of the top-down signal is spatially global. To study time, we varied stimulus duration and found that the perceptual system becomes gradually dominated by top-down information very early in the processing pipeline (Fig 6H, see also inset), to the extent that the initial dynamics of top-down control over bottom-up information may be sufficiently fast (< 30 ms) to approach the limit of reliable empirical characterization using psychophysical methods (see S1 Text for detailed analysis of the bottom-up effect suggested by the black trace in Fig 6H at 10–20 ms). These limitations are exacerbated by known conceptual difficulties with the interpretation of stimulus duration as reflecting processing time [57], whether in the presence or absence of a postmask (see S1 Text for detailed justification of the deliberate choice to avoid a postmask for the duration experiments [58, 59]). In the next section, we gain further insight into the ultrafast range via electrophysiological measurements.

### Electrophysiological markers of bottom-up/top-down effects

**Fig 7:**
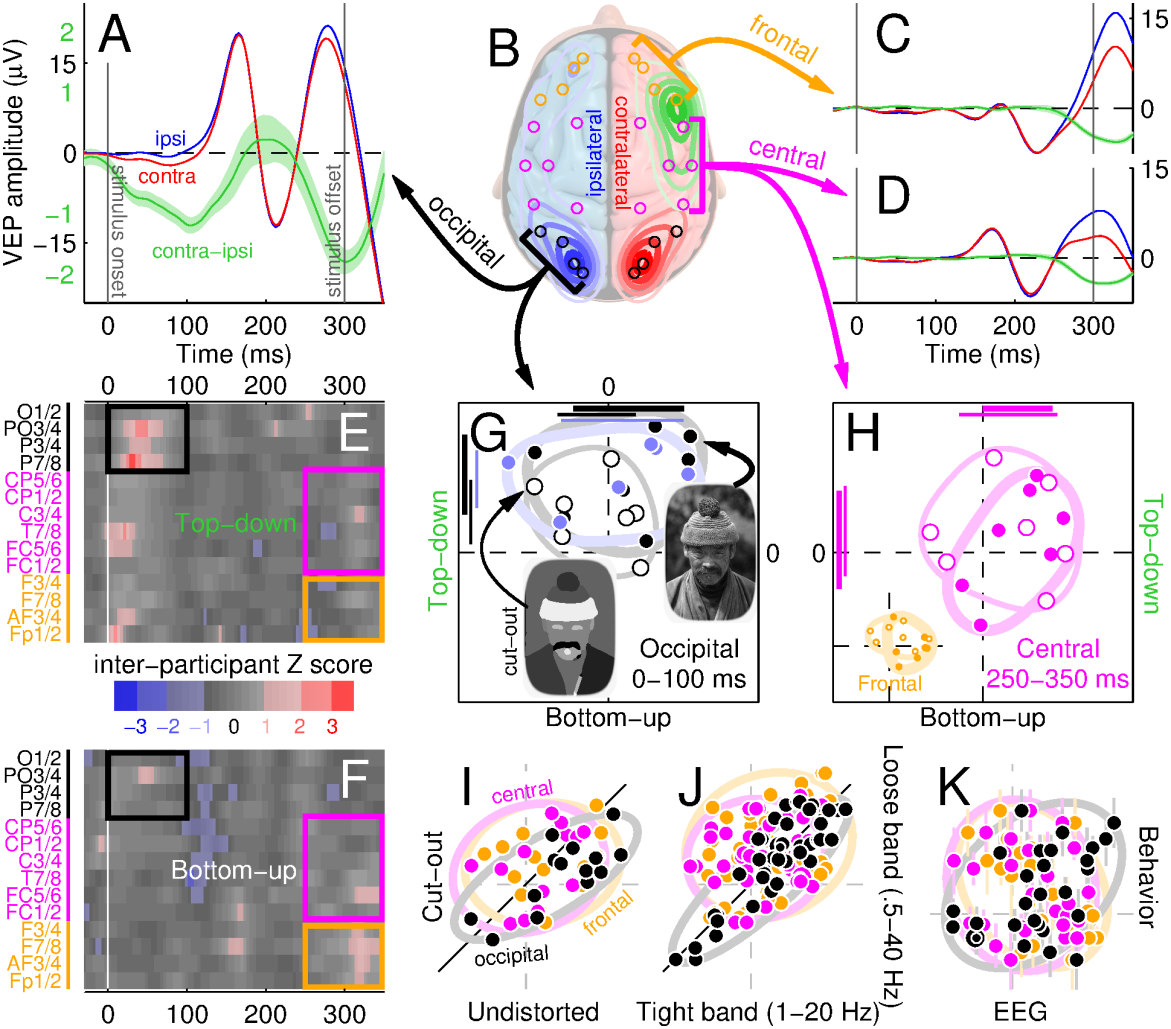
Top-down effects operate quickly within occipital cortex. **A**,**C**,**D** plot evoked potentials from occipital, central, and frontal electrodes marked by black, magenta, and orange circles in **B**. Blue/red trace shows waveform from electrodes ipsilateral/contralateral to probe location; green trace shows contralateral-minus-ipsilateral difference (shading shows ±2 SEM). Contour plots in **B** show interpolated scalp distribution of potential RMS for ipsilateral/contralateral waveforms (blue/red), as well as the ratio between contra-minus-ipsi waveform RMS and overall (ipsi + contra) RMS (green). **E**-**F** show the difference between rich and poor probe insertions for contra-minus-ipsi vaweform with respect to top-down (**E**) and bottom-up (**F**) maps, separately for the different electrodes (indexed on the *y* axis as pairs from which individual rows were computed), in the form of *Z* scores across participants. **G** plots RMS-normalized modulations (see Methods) in **E**/**F** on *y*/*x* axes pooled within black rectangles (occipital electrodes) in **E**/**F**, separately for different participants (1 symbol per participant, conventions similar to Fig 2F); solid symbols refer to intact scenes, open symbols to cut-out variant, blue symbols to results following artefact rejection (see Methods). **H** plots similar results from modulations pooled within magenta rectangles (central electrodes) in **E**/**F**; inset to **H** from modulations within orange rectangles (frontal electrodes). **I**-**K** plot the pooled quantities in **G**-**H** for specific comparisons on *x*- versus *y*-axes (top-down and bottom-up values are collated without distinction for this analysis): intact (undistorted) scenes versus cut-out variant (**I**); highpass/lowpass filtering of 1/20 Hz versus 0.5/40 Hz (**J**); values for intact scenes versus d′ log-ratios from Fig 2F (**K**). Ovals in **I**-**K** are aligned with best-fit line, with axes matched to 2 SD for values projected onto axes parallel/orthogonal to line. Data for this figure is available from S2 Data. EEG, electroencephalogram; RMS, root-mean-square; VEP, visual evoked potential.

The evidence presented above exposes signatures of a process that is spatially delocalized (Fig 6F and 6G) but highly localized in time (Fig 6H and 6I). The appropriate neuroimaging tool for probing these characteristics is the EEG with associated VEP [44]. Unsurprisingly, the bulk of EEG activity was measured from the occipital electrodes (black circles in Fig 7B) in both hemispheres (blue/red contours). Our focus is not on the VEP per se, but on the probe-specific component of the VEP; for this reason, we render the analysis probe-selective by computing the difference between the VEP contralateral to probe location (red contours) and its ipsilateral counterpart [46] (blue contours). Fig 7A plots the time course of these two waveforms pooled across the occipital electrodes, alongside their difference (green trace). With simple multistimulus arrays, the occipital contra-minus-ipsi waveform has been termed negativity 200-ms posterior contralateral (N2pc) [46] or posterior contralateral negativity (PCN) [60]. The green waveform we measure here must bear some relationship to these lateralized evoked potentials; however, our stimulus consists of a complex natural scene (as opposed to stereotyped search arrays [46, 60]), so that it is not necessarily the case that we should observe measurable differences between contralateral and ipsilateral waveforms. The fact that we do observe an idiosyncratic difference specific to a probe embedded within a natural scene is, in itself, a noteworthy result.

The amplitude of the difference waveform is nearly 1 order of magnitude smaller than the original waveforms (compare scaling of *y* axis for green versus black labels in Fig 7A), and modulates primarily within 2 time epochs roughly corresponding to 50–150 ms and 250–350 ms. To gauge the relative amplitude of the difference waveform against the amplitude of the original waveforms across the scalp, we plot a related quantity in Fig 7B (green contours). In relative terms, the difference waveform is most pronounced within central (magenta) and frontal (orange) electrodes. When we examine the time course of the relevant waveforms for these 2 electrode regions, however, we find that the difference waveform modulates only within the late phase (green traces in Fig 7C and 7D). Therefore, although the difference modulation is comparatively larger within central/frontal than occipital electrodes, the bulk of this modulation happens around stimulus disappearance, possibly reflecting offset responses and/or decisional/memory processes [61, 62]. In contrast, the difference waveform returned by occipital electrodes presents an early modulation more consistent with previous measurements of sensory-related activity [63] and clearly connected with visually-specific responses to stimulus information [64].

Based on the above observations, we focus our subsequent analysis on the 2 EEG processes that appear to dominate the electrophysiological measurements: an early process occurring within the occipital region (Fig 7A), possibly connected with what has been termed N1pc in previous studies [65], and a later process occurring within the central/frontal region (Fig 7C and 7D), possibly connected with the sustained posterior contralateral negativity (SPCN) waveform [61] (both may be at least partly connected with the N2pc in light of its potentially multicomponent nature [66] and variable latency [67]). Because our interest is in differential poor/rich effects for probe insertion, we compute contra-minus-ipsi waveforms separately for rich and poor locations, subtract rich from poor, and plot the result across all electrodes and time points for both top-down and bottom-up maps in Fig 7E and 7F respectively. These modulations are, therefore, 2 steps removed from the VEP: first by taking the difference between contralateral and ipsilateral waveforms to expose probe-specific effects [64], and further by taking the difference between rich and poor locations to expose the differential impact of bottom-up/top-down maps.

The only region where rich/poor differential effects display robust intersubject consistency (large *Z* scores) is indicated by the black rectangular outline in Fig 7E, corresponding to the early epoch (0–100 ms) of occipital activity. There appear to be other modulations within the surface plots; however, they are not robust and are unlikely to reflect relevant processes (see below). Based on our previous observations from Fig 7B–7D, we consider the 2 additional regions indicated by magenta/orange rectangles, corresponding to the late epoch (250–350 ms) of central/frontal activity. To evaluate occipital activity quantitatively, we sum modulations within the black rectangles from both bottom-up (Fig 7F) and top-down (**E**) descriptors, and plot them on *x* and *y* axes respectively in **G** (1 symbol per observer). Similarly to the behavioural effects (black symbols in Fig 2F), these measurements present a top-down effect with no bottom-up effect (black solid symbols fall above horizontal dashed line at *p* < 0.02 and scatter around vertical dashed line at *p* = 0.37). When we sum activity within the magenta rectangles for central electrodes, the resulting measurements display neither effect (solid symbols in Fig 7H scatter around the origin with no significant (*p* > 0.05) departures from the dashed lines). A similar result is obtained for frontal electrodes (inset to Fig 7H).

In additional experiments, we replicated the above effects using manipulated cut-out scenes (open symbols in Fig 7G) for which we had determined that the behavioural top-down effects also survived (blue data in Fig 3I). More specifically, differential effects for cut-out scenes (*x*/*y* values from Fig 7G) are strongly correlated across observers with those for undistorted scenes (r value is 0.76 at *p* < 0.002) only for occipital electrodes (black symbols in **I**), not for other electrode clusters (see S1 Text); the two datasets come from independent experiments, providing clear evidence that the adopted analysis/metric exposes genuine structure in the EEG. Furthermore, this structure is related to the perceptual effects, as evidenced not only by the similarity between electrophysiological and behavioural patterns (compare Figs 2F with 7G) but also by the significant correlation (r = 0.45, *p* < 0.02) between EEG and psychophysical markers for occipital electrodes in Fig 7K. Finally, we established that the EEG effects remain measurable when relevant aspects of the analysis are modified, such as choice of low/high-pass cut-off frequencies (Fig 7J) and exclusion/inclusion of common artefacts (see S1 Text).

We conclude that scalp signals originating from occipital cortex are modulated by neural constructs connected with the top-down map. These modulations become measurable very quickly (approximately 50 ms after stimulus onset), reflect the behavioural measurements (Fig 7G and 7K), generalize across independent experiments (**I**) and are not restricted to narrow filtering specifications (**J**). These measurements do not support more precise estimates of the timescale involved, not least because the exact figures will depend on several (and to a large extent arbitrary) constraints on the relevant analysis (e.g. which specific electrodes, filtering regime and others). The relevant region within Fig 7E (indicated by black rectangular outline) presents substantial modulations (reflected by red tint) between 30 ms and 70 ms. More accurate estimates of the timescale involved will require further EEG investigations combined with relevant single-unit measurements [53, 68].

### Sensitivity is enhanced by orientation retuning, not internal noise reduction

We have extensively documented that human sensitivity for performing probe discrimination is enhanced when the probe is inserted at rich locations on the top-down map. These effects are large (approximately 2× with mean enhancement across observers of an added 83%), dissociated from response criterion shifts (S3 Fig), and easily measurable: when 121 independent log-ratio estimates are combined from all experiments that individually showed statistically measurable top-down effects, the aggregate dataset is significant (different than 0) at *p* < 10^−20^ with 99.9% confidence interval of 0.43–0.63, meaning that the existence of the top-down effect is beyond doubt. Furthermore, this effect can be measured in the form of correlated scatter between sensitivity and top-down value, demonstrating that the performance enhancement tracks top-down information in a proportional fine-grained fashion (see S1 Text). Sensitivity measurements by themselves, however, do not impose sufficient constraints on possible sources of improved discrimination to allow conclusions about potentially underlying mechanisms [69].

**Fig 8:**
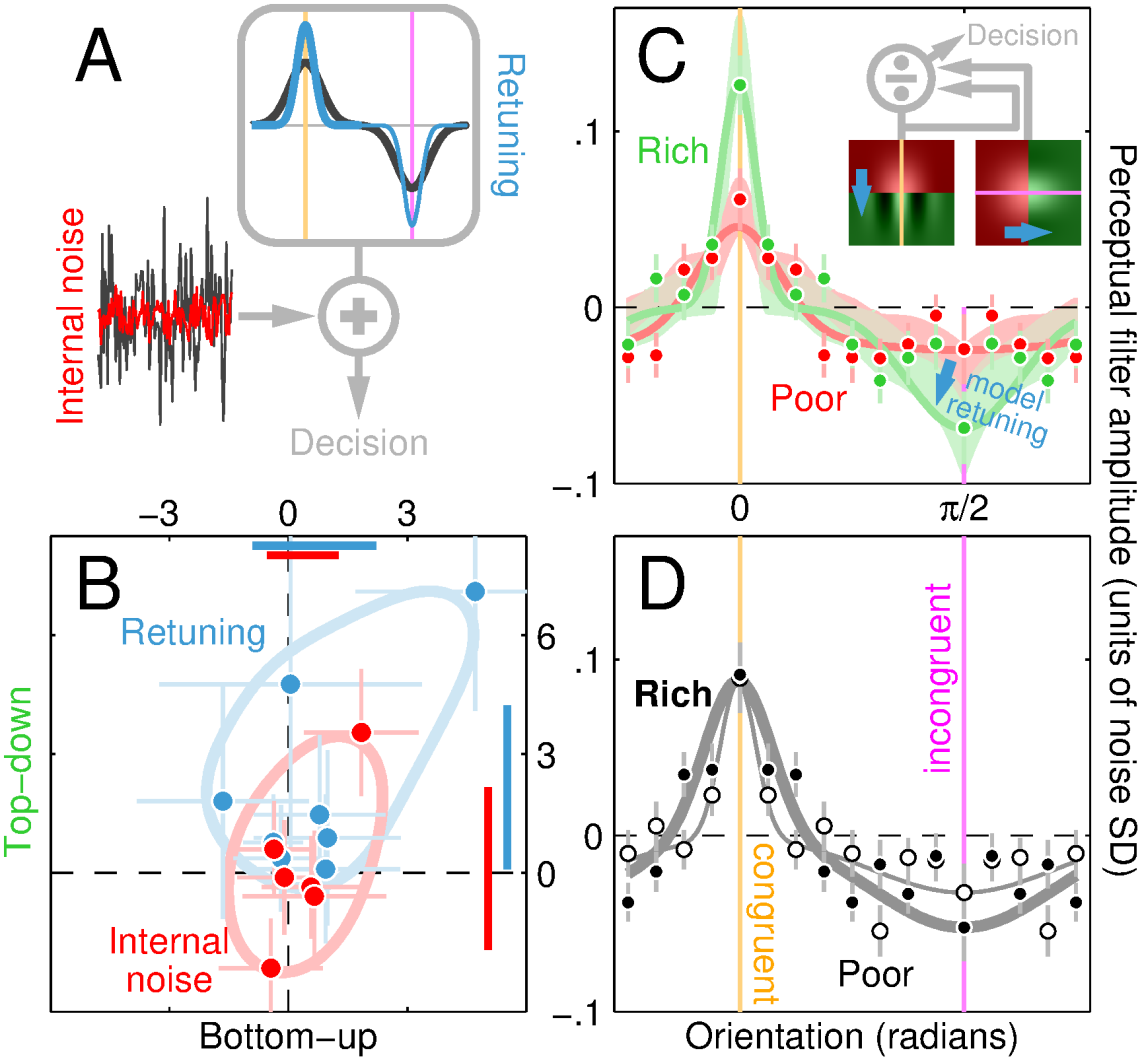
Top-down enhancement is driven by sensory retuning. **A** sketches minimal SDT model consisting of front-end filter (grey box) followed by additive internal noise (black random trace pointing to + symbol); sensitivity may be enhanced by reducing internal noise (red trace), sharpening filter around congruent (thick blue line) and/or incongruent orientation (thin blue line). **B** plots rich/poor log-ratios for internal noise estimates (red) and projected sensitivity from filter estimates (blue) returned by psychophysical reverse correlation (plotting conventions similar to Fig 2F). Aggregate perceptual filters are shown in **C**-**D** for rich vs poor locations on top-down (**C**, green versus red) and bottom-up (**D**, solid versus open) maps. Congruent/incongruent orientations are indicated by orange/magenta vertical lines (0 and *π*/2 on *x* axis). Error bars show ±1 SEM. Lines show fits from 2 Gaussian functions of opposite sign centred on congruent/incongruent orientations (for visualization only). Shading in **C** plots ±1 SD across simulations from gain-control model (inset), consisting of 2 front-end filters oriented along congruent (left icon in inset) and incongruent (right icon) orientations. Model simulations for red/green shading were generated by red/green-tinted front-end filters (transition indicated by blue arrows). Data for this figure is available from S2 Data. SDT, signal detection theory.

If we adopt a minimal SDT model [35] (Fig 8A) whereby orientation energy within the probe is processed by an orientation-selective filter [8, 70], a source of intrinsic variability is added to the filter response [37], and an output binary decision is produced [71], there are 3 fundamentally distinct ways in which the sensitivity of this mechanism may be enhanced: 1) by reducing its internal noise (red in Fig 8A); 2) by sharpening filter tuning around the congruent signal (blue thick line in **A**); 3) by sharpening filter tuning around the incongruent signal (blue thin line). We sought to determine which of these alternatives apply. To estimate internal noise, we performed additional experiments using an established double-pass methodology [36, 40] (see Methods). The resulting estimates (0.64/0.84/1.38 at 5/50/95 percentiles) fall within the expected range [37]; more importantly, they are not modulated by probe location along either the top-down or bottom-up map (red data points in Fig 8B scatter around origin).

To estimate orientation tuning of the perceptual process, we exploited a psychophysical variant of reverse correlation [31, 34] applied to the orientation noise injected into the probe. In line with previous work using isolated probes [8], the retrieved orientation-tuning functions peak at the congruent orientation (indicated by orange vertical line in Fig 8C and 8D) and present a negative modulation at the incongruent orientation (indicated by magenta vertical line). This characteristic remains unchanged when probes target rich as opposed to poor locations on the bottom-up map (compare solid with open data in **D**); however, it undergoes substantial retuning along the top-down map: at rich locations, tuning is sharper around both congruent and incongruent orientations (compare green with red data in **C**). To quantify these effects and make them directly comparable to the performance measurements, we compute the expected sensitivity associated with the shape of individual tuning functions and plot it as log-ratios in Fig 8B (see Methods). We observe a sizeable top-down effect (blue symbols fall above the horizontal dashed line at *p* < 0.01) without any bottom-up effect (blue symbols scatter around vertical dashed line at *p* = 0.46), mirroring the effects produced by direct sensitivity measurements (Fig 2F).

We conclude that the sensitivity enhancement associated with rich locations on the top-down map is the outcome of sensory retuning [10, 72] and not internal noise reduction, in line with other aspects of sensory processing [69, 73]. Further, our data demonstrate that retuning occurs at both congruent and incongruent orientations (thick/thin blue lines in Fig 8A). Retuning of this kind can be modelled by a physiologically plausible circuit such as the gain control operator [11] in the inset to Fig 8C. More specifically, small changes to the parameterization of this minimal model (indicated by blue arrows, see Methods) produce orientation-sharpening effects that closely match those exposed by data (model predictions are shown by green/red shaded regions).

### Deep networks generate proxy top-down maps

**Fig 9:**
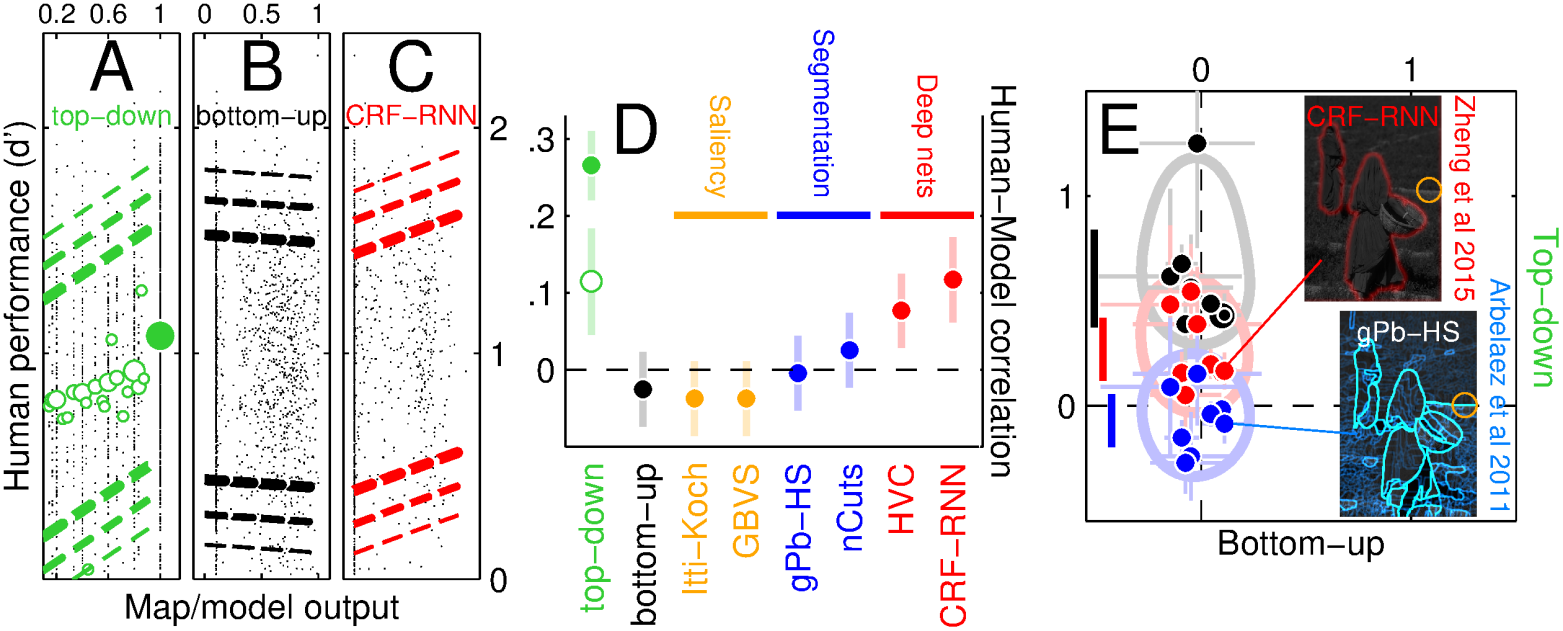
Deep networks generate good proxy for top-down representation. **A**-**C** plot human sensitivity (y axis) for individual probe insertions (one small dot per insertion) separately for different scenes (pooled across participants), against values corresponding to probe insertion point on top-down/bottom-up maps (**A**/**B**) and the map generated by the CRF-RNN deep convolutional network [23] (**C**; abscissa values for this plot have been rescaled to range between 0 and 1). Dashed lines show 80%, 90%, and 95% (from thick to thin) confidence intervals for linear fit. Green symbols in **A** show average *y* value for individual abscissa values; symbol size scales with number of data points. **D** shows correlation values for scatter plots in **A**-**C** and those generated by other computer vision algorithms (Itti-Koch [3], GBVS [41], gPb-HS [5], nCuts [42], HVC [43]); open green symbol plots correlation for top-down map when consensus probe locations (indicated by solid green symbol in **A**) are excluded. Error bars in **D** show 95% confidence intervals. **E** plots rich/poor log-ratios to the conventions of Fig 2F where human sensitivity estimation for *y* axis is relabelled against rich/poor probe locations on the maps generated by CRF-RNN (red) and gPb-HS (blue) algorithms instead of top-down map (black). Values on the *x* axis are computed with respect to bottom-up map (same as Fig 2F). Icons show example segmentations from the two algorithms for the natural scene in Fig 1A; coloured overlay indicates segmented regions/boundaries, orange circle corresponds to red solid circle (top-down poor, bottom-up rich location) in Fig 1D. Data for this figure is available from S2 Data. HVC, hierarchical visual cues; GBVS, graph-based visual saliency.

What is the potential origin of the signal driving the circuit transition in Fig 8C and the associated sensitivity enhancement? We implemented a selection of computer vision algorithms (see Methods) to determine whether the resulting scene representations correlate positively with human sensitivity as observed along the top-down map (Fig 9A; see S1 Text). There is no such correlation when sensitivity is similarly plotted against the bottom-up map (r = −0.02, *p* = 0.3, Fig 8B) or established saliency/segmentation algorithms (orange/blue in Fig 9D); however, last-generation deep networks (red) generate map values that correlate significantly with human sensitivity (see also Fig 9C). We focus on a recent deep convolutional network (DCN) for semantic segmentation [23] (CRF-RNN) that is able to achieve a correlation value comparable to that returned by nonconsensus values on the top-down map (open green symbol in Fig 9D). When sensitivity log-ratios from individual observers are computed with relation to the probe rich/poor classification generated by the DCN, we observe a nontrivial top-down effect (red data in **E**, *p* < 0.01) not captured by established segmentation algorithms [5] (blue data). The DCN seems able to exclude physically rich locations in the image that are not perceptually interesting (examples are indicated by yellow circles in Fig 9E), while this task remains challenging for some other computer vision algorithms [6]. Indeed, it has long been recognized that this is one of the core unresolved issues in vision science [2].

## Discussion

The main experimental result of this study can be summarized by the notion that local visual operators are controlled by the segmented layout implied by the physical image, rather than by the physical image itself. This result should not be interpreted to mean that the physical content of the image is entirely irrelevant: in the trivial limit-case when image contrast is reduced below detection threshold, the scene becomes invisible and no visual processing can take place. Our results indicate that, provided the physical content of the image is minimally sufficient to support inference of the underlying environmental structure, the latter process becomes the primary contextual influence on image reconstruction, overriding strictly image-driven aspects of visual processing (e.g., collinear facilitation [74]). These findings suggest that once the inferential mechanism is kick-started, perception is quickly organized around the operation of this mechanism [75, 76]; detailed variations of physical content are then sidelined by the object segmentation projected by the inferred layout of the scene. The behavioural impact of this process can be measured in the form of a top-down effect on perceptual efficiency for local image reconstruction. We speculate that object segmentation is actively engaged at all times; in the laboratory, it may go unnoticed unless experiments are specifically designed to expose its impact on feature extraction.

At a cursory level, it may appear that the top-down effect simply reflects known phenomena such as spatial uncertainty [77], crowding [78], flanker facilitation/inhibition [12], texture (second-order) cues [79], and eye-movement scanning strategy [80]. However, we can exclude these factors by evaluating them against the wide-ranging collection of our experimental results (see S1 Text for detailed argumentation relating to these and other factors). For example, top-down effects show no dependence on eccentricity (S2 Fig) and cueing of probe location (Fig 2G), the former inconsistent with crowding [81] and the latter inconsistent with a role for spatial uncertainty. We must conclude that the phenomena exposed by our probe-insertion paradigm cannot be accounted for by commonly proposed mechanisms and reflect a genuinely novel class of perceptual processes unexplored previously. Past literature has established that object identity is represented as quickly as approximately 100 ms after the visual stimulus has appeared [82, 83]; however, little is known about the perceptual operations that lead up to said representation [84], and that must occur during those initial 100 ms [85]. The present results speak to the nature and timescale of those operations and their relationship to natural scenes.

In our prior research with embedded probes, probe insertion was restricted to one specific location for each natural scene [9, 10], thus providing no useful information relating to the topics addressed in the present study. Instead, it focused on the effect of image inversion, a manipulation believed to selectively target semantic representations [52], and found that it may impact the structure of perceptual filters with no concomitant change in discrimination performance [9, 10]. In apparent contrast, the top-down effects we report here involve marked improvements in sensitivity. These apparently conflicting results owe their distinct patterns to the different stages/mechanisms probed by the 2 different sets of experiments. Image inversion has no impact on the top-down effects that represent the focus of this investigation (red symbols in Fig 2F), and overall performance was no different between upright and inverted trials (*p* = 0.64), just as in the earlier work [9, 10], and in line with related single-unit measurements [53]. It is clear that the representational stage targeted by manipulating probe location is distinct from the stage interrogated by scene inversion [52]. We speculate that the current experiments probe a stage corresponding to the segmented image where object boundaries are delineated and objects possibly segregated [53, 68, 85], without necessarily assigning semantic content to the segmentation. This concept builds on the distinction between tracing out an object from a scene on the one hand and knowing what that object is on the other hand [2, 21, 68, 86]. We propose that only the former operation is probed effectively by the image manipulations adopted in this study.

The above distinction is critical for correctly situating our electrophysiological results in relation to those associated with ultrafast image recognition [82]. In those classic studies, the earliest EEG signatures of image recognition occur at approximately 100 ms [82, 87], after the top-down modulation in our data has completed (Fig 7E). We speculate that our experiments probe the segmentation stage immediately preceding scene recognition [2, 16, 53, 68, 88], and in doing so expose the temporal evolution of different phases in the perceptual reconstruction and interpretation of natural scenes [85]. In this sense, our study is not only compatible with classic results from the ultrafast recognition EEG literature [87] but also provides novel and distinct information about the underlying mechanisms that has not been exposed by those previous studies. Furthermore, it is entirely consistent with spike measurements from single neurons in the primary visual cortex [53, 68, 85]. Those measurements have identified at least three stages in image processing: detection, segmentation, and attention, unfolding in temporal succession at approximately 50, 60, and 140 ms after stimulus onset [85]. The effects exposed here naturally speak to the second stage: they must originate beyond detection but before attentional deployment (Fig 2G). Consistent with this interpretation, their EEG dynamic characteristics dovetail the single-neuron measurements [53, 68, 85] (although the link can only be tentative at this stage, given innumerable differences in the adopted stimuli, such as artificial boundaries defined by motion [85] as opposed to static boundaries defined by natural scenes used here).

In contemporary accounts of natural image understanding, this process is almost invariably connected with the notion of feedback and top-down signals [20, 89], informing our own choice of map labels and associated probe-insertion protocol (Fig 1). It is unclear, however, whether the bottom-up/top-down distinction [28] represents the most productive conceptual framework for understanding the results presented here [29, 30]. We find that electrophysiological signatures of top-down effects become measurable shortly after stimulus onset [53, 68] (Fig 7E); furthermore, they are restricted to occipital cortex (Fig 7G). This result, combined with stable behavioural counterparts at very short stimulus durations (Fig 6H and 6I), indicates that the perceptual system is dominated by object segmentation from immediately after stimulus presentation throughout the subsequent 100-ms epoch [50, 63]: there is little in our dataset that points to a feedback mechanism as typically conceptualized in the literature [28, 89]. For example, allowing the scene to be analyzed only after the probe has already disappeared, a manipulation expected to impact our measurements [90, 91] under feedback accounts [20, 89], does not reduce top-down effects (Fig 5G). We propose that the process involved in these experiments is best understood as an integrated module where the distinction between bottom-up and top-down processing is not transparently attached to identifiable submodules [29, 63, 92–94].

If we accept the notion of an integrated module [88, 92], the processing mode engaged by the perceptual system reflects a sensitivity bottleneck [95, 96], rather than absence/presence of top-down feedback. The clearest dissociation between top-down-dominated and bottom-up-dominated processing modes is offered by intact scenes on the one hand, and blurred (lowpass) scenes on the other (black and yellow symbols occupy extreme positions along negative diagonal direction in Fig 4). We propose that the perceptual system operates all along in a manner that depends on both types of information contained within the two maps provisionally labelled “top-down” and “bottom-up;” however, the bottleneck for performing local image reconstruction is defined by the former kind of information in the presence of an intact interpretable scene (Fig 2F), while it is defined by the latter kind of information in the presence of a degraded unintelligible image (Fig 3B and 3C).

The above notion is not meant to challenge the wider applicability of feedback and top-down control [28, 89]: our own dataset displays characteristics suggestive of a general role for feedback, such as the impact of scene-probe temporal asymmetry on asbolute sensitivity (blue symbols in Fig 5E). Rather, the notion of an integrated module is intended in a restricted and specific sense. First, as discussed above, it only applies to the processing stage probed by the manipulations investigated here, which we have provisionally described as the segmented representation of the scene [16, 22, 23]; second, it relates to perception, not to the underlying anatomy or physiology: it is conceivable that the neural implementation may involve a default network of early visual areas [53, 68, 88, 97, 98] communicating in a fashion that may be characterized as feedback [99, 100], although on a faster timescale than typically associated with top-down control [63, 88, 101, 102]. Our results demonstrate that the visual process of reconstructing meaningful boundaries from natural scenes immediately engages such integrated extraction/segmentation perceptual module [29, 53, 68, 88, 92, 101, 103], the operation of which is not dependent upon attentional deployment [10, 104, 105] (Fig 2G), relies on various statistical properties of the scene (Fig 4), extends over large spatial scales [76, 98] (Fig 6A–6G and S2D Fig), resides within occipital cortex [85] (Fig 7G), and retunes its machinery to hone into the expected signal without changing its intrinsic variability [13, 54, 69, 73] (Fig 8B). It is feasible to construct a computational support for this integrated architecture based around plausible [11] and primarily feedforward (i.e., fast) neural networks [106] (Fig 9C–9E). Future research will be necessary to characterize the biological circuits that support this process [107] and establish their connection with the perceptual phenomena which we have documented here [108].

## Supporting information

S1 Text Document providing additional information and detailed discussion of specific issues. (PDF)

S1 Fig Orthogonality of bottom-up/top-down maps. Value on top-down map (*y* axis) is plotted against value on bottom-up map (*x* axis) across all probe insertions (1 symbol per insertion); open/solid indicates poor/rich on bottom-up map, red/green indicates poor/rich on top-down map. Marginal distributions along the bottom-up map (top histograms) are virtually identical for poor/rich locations on top-down map (red/green solid histograms); similarly, marginal distributions along the top-down map (right histograms) are indistinguishable for poor/rich locations on bottom-up map (open/solid histograms). Standard correlation tests are not applicable because this dataset is not normally distributed (Henze-Zirkler test) and it is heteroscedastic (test based on conditional variances).
(TIF)

S2 Fig Visual field distribution of probe insertions and discrimination performance. Probe density declines with eccentricity on both bottom-up (**A**) and top-down (**B**) maps (see overall decreasing characteristic of plots); in both cases, there is little difference between poor and rich insertions (open/solid in **A**, red/green in **B**; smooth lines show polynomial 2-degree fits), although there appears to be a moderate trend for rich insertions to exceed poor insertions near the fovea, and poor insertions to exceed rich insertions at 6–8 degrees of eccentricity (see blue trace plotting rich/poor log-ratios; shading shows ±1 SEM). Human sensitivity also declines with eccentricity as expected [109] (overall decreasing characteristic in **C**-**D**), but it displays different trends for poor/rich differential effects: no difference between poor and rich insertions on the bottom-up map at any eccentricity (**C**), and clear differences on the top-down map at all eccentricities (**D**). Error bars show ±1 SEM.
(TIF)

S3 Fig Criterion shifts associated with enhanced sensitivity. **A** shows ROC plot [35] of individual data (1 symbol per observer) pooled across conditions that showed a top-down effect without bottom-up effect, for bottom-up poor/rich (black open/solid) and top-down poor/rich (red/green) insertions. Solid lines show best-fits of equal-variance SDT model for variations of sensitivity (d′), dashed lines in inset to **A** show fits for variations of criterion c; gray/black lines refer to bottom-up poor/rich data, red/green to top-down poor/rich data. Inset magnifies top-down rich/poor data clusters with associated d′/c fits. **B** plots d′ against c computed under the equal-variance assumption for all data points in **A**; the 2 quantities are clearly correlated. Error bars show ±1 SEM. Solid line shows best linear fit, dashed lines show 95% confidence intervals for fit. **C** plots rich/poor log-ratios computed from both d′ (*y* axis) and c (*x* axis) with reference to bottom-up (black) and top-down (green) maps; segments near *x*/*y* axes show 95% confidence intervals around mean values and demonstrate that the top-down fractional effect for sensitivity (green vertical segment near right *y* axis) is much greater than the effect for criterion shifts (green horizontal segment near top *x* axis). Axes in **C** have been scaled to match for direct comparison. ROC, receiver operating characteristic.
(TIF)

S1 Video Example sequence of 10 trials for main condition (unperturbed natural scenes except for probe insertion). In this demo, the first trial is an example of “postcue” trial where the spatial cue (bright blob indicating probe location) appears after the natural scene, the second trial is an example of “precue” trial (cue appears before scene), and subsequent trials alternate between postcue and precue. In the actual experiments, trials were randomly assigned to precue or postcue categories (i.e., there was no regular repeating sequence; the postcue-precue repeating sequence was adopted in this demo for clarity of exposition).
(AVI)

S2 Video Example sequence of 8 trials for the “zooming” condition where the stimulus involves a smooth transition between a probeless scene and a sceneless probe. In this demo, the first trial shows an example of “zoom-in” (scene-to-probe) transition at longer (300-ms) duration, the second trial shows an example of “zoom-out” (probe-to-scene) transition at longer duration, the third trial shows an example of “zoom-in” transition at shorter (100-ms) duration, the fourth trial shows an example of “zoom-out” transition at shorter duration. The subsequent 4 trials repeat this sequence. In the actual experiments, trials were randomly assigned to zoom-in/zoom-out and long/short categories (i.e., there was no regular repeating sequence; the zoom-in/zoom-out and long/short repeating sequence was adopted in this demo for clarity of exposition).
(AVI)

S1 Data Data dump. (ZIP)

S2 Data Figure data dump. (ZIP)

## Abbreviations

DCN: deep convolutional network
EEG: electroencephalogram
N2pc: negativity 200-ms posterior contralateral
PCN: posterior contralateral negativity
SDT: signal detection theory
SPCN: sustained posterior contralateral negativity
VEP: visual evoked potential
