# Supplementary material for: Object segmentation controls image reconstruction from natural scenes
Source: PLoS Biol. 2017 Aug 21;15(8):e1002611. doi: 10.1371/journal.pbio.1002611 (PMC5565198; doi:10.1371/journal.pbio.1002611)
Supplement: S1 Text — (PDF) [file pbio.1002611.s001.pdf]

S1 Text for 'Object segmentation controls image reconstruction from natural scenes'

Author name: Peter Neri
Author affiliation: Laboratoire des Systèmes Perceptifs, Département d'études cognitives, Ecole Normale Supérieure, PSL Research University, CNRS, 75005 Paris, France

1 Cognitive factors

1.1 Top-down effects do not require semantic processing

If we provisionally consider hierarchical accounts of visual processing [1], ranging from low- [2] through to mid- [3, 4] and higher-levels [5, 6], the rich-vs-poor differential effect on the top-down map must originate from a stage that is above low-level (due to the orthogonal construction of the top-down map with respect to low-level image content, see **Supplementary Figure 1**). This observation places a lower bound within the hierarchy, however it is unclear what the upper bound would be: the relevant stage may lie anywhere above low-level, whichever hierarchical framework is adopted. For example, we may subscribe to a minimal three-stage pipeline [7, 8] where images are first encoded as disconnected collections of local features (stage 1), those features are then grouped according to the inferred structure of object segmentation (stage 2), and finally segmented objects are semantically labelled as 'people' or 'buildings' (stage 3). Within this framework, the top-down effect in **Figure 2E** may originate from either stage 2 or stage 3. More information is needed to determine which one of these two alternatives may be applicable.

We selectively disrupted the semantic stage (termed 3 above) via the established manipulation of image inversion: when flipped upside-down, meaningful images (such as faces or scenes) become more difficult to interpret correctly [9–11]. If the top-down effect in **Figure 2E** originates from the semantic representation (stage 3), this effect should be reduced (possibly eliminated) by upside-down inversion [12, 13]. We tested this prediction directly by simply repeating our sensitivity measurements with inverted images, and found that the role of semantics is *not* supported by data: the top-down effect was comparable to that obtained with upright images in both size and statistical significance (compare confidence intervals indicated by red/black vertical segments near y axis in **Figure 2F**). This finding sets an upper bound on the origin of the top-down effect at a level preceding the semantic stage, possibly corresponding to what we termed stage 2 (segmentation stage) in the simplified hierarchical description. Combined with the lower bound defined above, this additional result enables enough specification of the relevant phenomenon to support clear connections with existing literature (see Discussion, main text). We found similar results with contrast-reversed scenes (not shown), further confirming the lack of semantic involvement (contrast reversal is widely regarded to achieve a similar goal to image inversion in disrupting semantic representations [14]).

1.2 Top-down effects are orthogonal to spatial attention

Previous literature has pointed to spatial attention as potentially playing a key role in natural scene understanding [15–17], although the exact nature of its impact remains unclear [18–22]. Prompted by these findings, we manipulated attentional deployment in an effort to determine its role (or lack thereof) in driving the top-down effect. On 'precue' trials, the natural scene was preceded by a bright blob centred on the location subsequently occupied by the grafted probe, thus providing observers with a valid spatial cue; on 'postcue' trials, the cue only appeared *after* the natural scene (see Methods and **Supplementary Video 1**). Observers were therefore afforded the opportunity to deploy spatial attention to the probe on precue, but not postcue, trials [23, 24].

The cueing manipulation had no impact on the top-down effect (both black (precue) and magenta (postcue) symbols fall above horizontal dashed line in **Figure 2G** at $p<0.01$ with overlapping confidence

intervals). This lack of any measurable impact is not a result of observers simply ignoring the spatial cue (which they may have potentially chosen to do, as this strategy would not preclude performing the task): performance was substantially higher on precue trials (y axis in **Figure 2E**) as opposed to postcue trials (magenta symbols fall above equality line at $p<0.01$), unambiguously demonstrating that observers did in fact exploit the reduction in spatial uncertainty conveyed by the cueing manipulation [24] (see also confidence interval indicated by magenta diagonal segment in **Figure 2E**). We conclude that spatial attention boosts overall sensitivity, however it has no impact on the differential top-down modulation of sensitivity. This result can also be understood as indicating that attention does not change the qualitative nature of how image processing operates in relation to the assigned task: the effect of attention is orthogonal to the top-down manipulation, similar to other aspects of image understanding [25, 26].

2 Scene manipulations

2.1 Potential role of spatial frequency

The two fundamental attributes of static images are orientation and spatial frequency [27]; these two properties are prominently represented within the neural code [28], and can support adequate image reconstruction [29, 30]. Spatial frequency, for example, underlies coarse-to-fine models of image understanding [2, 31–33]. To investigate its potential role, we generated highpass and lowpass filtered versions of the natural scenes [34] (examples are shown in **Figure 3A** and **B** respectively). We did not alter the region immediately surrounding the probe because our goal is to selectively understand the role played by the global, not local, content of the scene (had we altered the region immediately surrounding the probe, we would be unable to tease apart local versus global effects on sensitivity). The top-down effect is retained with highpass scenes (blue symbols fall above horizontal dashed line in **Figure 3C** at $p<0.01$) but it is lost with lowpass scenes (red symbols scatter around dashed horizontal line at $p=0.19$; see also confidence intervals indicated by vertical segments near left y axis). It is relevant in this respect that the interpretability of highpass scenes is largely retained for our database, while it is almost invariably lost with lowpass scenes (due to heavy blurring, lowpass images do not deliver recognizable content). Interestingly, both manipulations present a bottom-up effect, particularly the lowpass manipulation: for the highpass case, the effect is mildly significant at $p<0.04$; for the lowpass case, it is significant at $p<0.01$. Given the presence of a data point (red symbol occupying bottom-right region of plot) that departs from the main cluster for the lowpass data, we applied outlier detection (Grubbs test) and indentified the abscissa value (bottom-up) as potentially outlying (at $p<0.05$). After removing this data point, the bottom-up effect remained significant at $p<0.02$. We also combined lowpass and highpass to test for collectively manipulating bandpass content, and found a significant bottom-up effect at $p<0.002$. All effects were also obtained with a more conventional (but less appropriate for this dataset) t-test. It is therefore clear that bandpass manipulations lead to bottom-up effects (nearly all data points in **Figure 3C** fall to the right of the vertical dashed line), while sometimes also retaining a top-down effect (highpass manipulation).

2.2 Potential role of orientation content

The above results indicate that image intelligibility may play a pivotal role in determing the top-down effect. To further study the role of scene interpretability, we developed a warping algorithm that gradually deforms image structure while largely retaining texture characteristics (**Figure 3D**-**E**). To a limited extent, this manipulation selectively targets orientation content while leaving spatial frequency relatively unaffected. Warping does not eliminate the top-down effect (blue/red symbols in **Figure 3F** fall above horizontal dashed line at $p<0.02$), however it reduces its size, and it does so progressively: mild warping (**Figure 3D**) returns a mean top-down effect of $\sim$0.5 compared with $\sim$0.6 for intact natural scenes, further reduced to $\sim$0.3 with strong warping (**E**). The different magnitude of top-down effect between mild and strong warping survives direct comparison (green data points in inset to **Figure 3F** scatter

above diagonal equality line at $p<0.04$).

2.3 Potential role of segmented regions/boundaries

As a further step in elucidating the role played by global image structure, we applied two drastic manipulations: in the 'cut-out' variant, segmented objects are filled with randomly assigned uniform intensity (**Figure 3G**); in the 'line' variant, only their contours are retained as bright lines within binary images (**Figure 3H**; see Methods). Perhaps surprisingly, the top-down effect is still measurable with cut-out scenes (blue symbols in **Figure 3I** fall above horizontal dashed line at $p<0.01$), but it is completely eliminated by the line manipulation (red symbols scatter around horizontal dashed line at $p=0.19$; see also confidence intervals indicated by vertical segments near y axis). It is relevant in this respect that most cut-out scenes retain intelligibility, while line-manipulated scenes almost invariably lose interpretability. Incidentally, these two manipulations exclude any role for two potential artefactual results, namely that top-down effects may be a result of differential probe distribution across the visual field and/or low-level second-order image cues that are not captured by the bottom-up map (see sections 6.1 and 6.2).

2.4 Potential role of phase/power spectra

Manipulations of phase/power spectra have previously been exploited to demonstrate that image intelligibility depends far more on the phase structure of the Fourier spectrum than on its amplitude [35]. In the 'phase-only' manipulation (magenta in **Figure 4**), the phase structure of the spectrum is retained while the amplitude (power) structure is replaced by a random perturbation; in the 'power-only' variant (cyan in **Figure 4**), the complementary manipulation is applied (see Methods). Although it was often difficult to obtain reliable measurements with such highly degraded images ('power-only' scenes consisting of no more than unintelligible texture), our results are consistent with the notion that image interpretability is supported primarily by the phase spectrum [35–37]: the top-down effect was retained with 'phase-only' scenes, while it was entirely eliminated with 'power-only' scenes.

3 Role of spatiotemporal characteristics

3.1 Spatiotemporal relationship between probe and scene

Feedback interpretations of top-down effects [38] may lead to the expectation that these effects should depend on the temporal order of information accrual from the probe in relation to the surrounding natural scene [39]. More specifically, if top-down effects reflect the operation of a scene-interpreting module that sends signals back to the local machinery in charge of analyzing the probe [40, 41], the time cost associated with signal transmission should mean that top-down modulation should be greater when the scene is presented *before* the probe as opposed to being presented *after* the probe [39]. We tested this prediction by designing 'zooming' variants of our stimulus, where a smooth transition was enacted between a probeless scene (left-most image in **Figure 5A**) and a sceneless probe (right-most image in **Figure 5A**). The zooming algorithm carries out this transition for any probe location and type (see examples for all 4 probe types in **Figure 5A**-**D**; see also Methods and **Supplementary Video 2**). We tested two zooming directions, 'zoom-in' (scene-to-probe moving rightward in **Figure 5A**-**D**) and 'zoom-out' (probe-to-scene moving leftward in **A**-**D**), as well as two different stimulus durations, short (entire zooming transition lasting 100 ms) and long (300 ms).

Similar to attentional deployment, we found that zooming direction/duration had a sizeable impact on overall performance (blue/red data points in **Figure 5E** fall above/below diagonal equality line at $p<0.01$), however the top-down effect was left unaffected by either manipulation (red/magenta/light-blue symbols in **Figure 5F**-**G** fall above horizontal dashed lines at $p<0.01$, blue symbols at $p<0.02$; see also confidence intervals indicated by vertical segments near y axes). More specifically in relation to zooming direction, the scene-to-probe transition (zoom-in, y axis in **Figure 5E**) was associated with

better performance than the opposite transition (zoom-out, x axis). We interpret this result as indicating that, although the spatiotemporal relationship between scene and probe does play a role in determining overall sensitivity for analyzing the probe and it does so in the direction of scene analysis facilitating probe analysis, the qualitative operation of the system in relation to the top-down/bottom-up distinction is orthogonal to spatiotemporal dynamics.

3.2 Role of space in isolation

The zooming stimulus compounds spatial with temporal manipulations. To selectively study the role of spatial layout, we introduced a gap of varying size between the probe and the surrounding scene (**Figure 6B**-**E**). Because probe discrimination was more challenging with large (**Figure 6E**) as opposed to small gaps (**Figure 6B**), we adjusted the signal-to-noise ratio (SNR) of the probe to ensure that overall performance was consistently above chance, and of comparable magnitude, for all gap sizes (red trace in **Figure 6F**). The gap had virtually no effect on top-down/bottom-up effects: the bottom-up effect was absent at all gap sizes (black symbols in **Figure 6F**), while the top-down effect was present at all gap sizes (green symbols).

To evaluate this observation more quantitatively using individual observer analysis, we notionally split gap sizes into 'small' (gap smaller than probe) and 'large' (gap larger than probe). The top-down effect was equally strong and robust for the two gap groups (blue/magenta symbols in **Figure 6G** fall above horizontal dashed line at $p<0.01/0.05$). We also observed a marginally significant bottom-up effect for large gap sizes in the direction of better performance for poor locations (magenta symbols fall to the left of vertical dashed line at $p<0.05$), however this effect is not confirmed by confidence interval estimation (magenta horizontal segment near top x axis overlaps with vertical dashed line) and has therefore likely arisen by chance. We conclude that the top-down effect has a global (spatially extended) origin, consistent with the notion that it reflects scene interpretation [34, 42].

3.3 Role of time in isolation

To isolate the role of time, we varied stimulus presentation between 10 and 300 ms; again, we adjusted probe SNR to ensure that performance was stable across the entire range (red trace in **Figure 6H**). We chose to avoid the use of a post-mask for three reasons. First, because there is no accepted mask design for natural scenes that can be safely adopted without complicating data interpretation [43, 44]. Post-masks do not simply 'interrupt' visual processing but rather interact with the stimulus in complex ways [43, 45, 46], and the nature of this interaction depends on the structure of the mask [47]. Adopting a post-mask may therefore disrupt the processing mode that would be engaged by the perceptual system during natural vision, and eventually distort data interpretation [43, 47]. Second, even if one were to assume that the post-mask does not distort visual processing (which is incorrect as explained above), relevant/critical processing can impact perception well after the mask has supposedly 'interrupted' visual processing, as now unequivocally demonstrated by both psychophysical [45] and EEG measurements [48]; in other words, there is no sense in which a post-mask can be used to restrict perceptual processing to a specific time period preceding the mask [47, 48]. Third, at the ultra-short stimulus durations required to observe a decrease of the top-down effect and the emergence of a bottom-up effect (10-30 ms), the presence of a post-mask reduces performance to such an extent that the protocol is no longer viable for testing with naive observers. We collected 800 trials ($\sim$200 trials per individual d' estimate) at 20-ms stimulus duration on a non-naive, extensively experienced observer (author PN), in the presence of a post-mask. The post-mask extended over the entire natural scene, was presented immediately after the stimulus, and consisted of pixel noise (each pixel was independently assigned a randomly selected luminance value from a uniform distribution spanning the whole monitor range). The probe contained a noiseless signal at $\sim$40% contrast (an optimal setting for supporting discrimination) and the stimulus was always preceded by a precue. The average d' value under these favourable conditions was merely $\sim$0.2, making this protocol inadequate for naive observer testing; we attempted data collection on the naive observer with highest performance efficiency within our participant pool, but she could not perform

above chance (d′ ∼0). Despite the extremely low performance achieved by the non-naive observer, it was still possible to measure *differential* effects in the form of rich/poor d′ log-ratios and confirm the results obtained without a post-mask (detailed in the next paragraph): the top-down effect survived at ∼1.5, together with the presence of a bottom-up effect at ∼0.5 (log-ratios). To summarize, the adoption of a post-mask was neither desirable [43, 46, 47] nor feasible for extensive data collection in the context of our experiments; the limited measurements we were able to obtain using a pixel-noise post-mask are consistent with those obtained without the use of a post-mask.

Although the top-down effect survives at most stimulus durations, it demonstrates a steady tendency to decrease at shorter durations (green symbols in **H**); this trend may not be evident due to the relatively small log-ratio range spanned by the top-down characteristic in **H** (green trace is compressed along y axis), but it is strong when assessed via correlation coefficient (Pearson's r=0.91 at p<0.005, 95% confidence interval 0.64-0.99; also significant in all tests from robust correlation battery [49]; data has been rescaled and replotted in inset to **H** to aid visualization of positive trend). Interestingly, the bottom-up effect shows a complementary pattern: as documented previously, it is absent for durations in the 100-300 ms range, but it becomes visible for very short durations (black symbols in **H**). To evaluate these trends in more detail, we group stimulus durations into short (less than 30 ms) and long (≥30 ms). At long durations, we measure a top-down effect with no bottom-up effect (magenta symbols in **Figure 6I** fall above horizontal dashed line at p<0.01 and scatter around vertical dashed line at p=0.94). Conversely, at short durations we measure a bottom-up effect with no top-down effect (blue symbols in **I** fall to the right of vertical dashed line at p<0.01 and scatter around horizontal dashed line at p=0.25). At this level of analysis, it appears that the system switches from bottom-up to top-down mode very quickly on a timescale of ∼30-50 ms, consistent with measurements reported for the extraction of colour information from natural scenes [50].

The above-detailed bottom-up→top-down transition must be interpreted with caution, due to the difficulty of obtaining reliable measurements at short durations: some observers were excluded from estimates at 10/20 ms because they could not perform the task (see Methods), and statistical significance assessed with p values is not entirely consistent with confidence intervals. More specifically, the 95% confidence interval for bottom-up effects at short durations (horizontal blue segment near top x axis in **Figure 6I**) includes 0 (no effect), casting doubt on the interpretability of the small p value returned by the sign-rank test for this effect. This discrepancy may originate from the presence of a potential outlier (blue data point at bottom-right, Grubbs test [51]): when this data point is removed, p values (0.22/0.03 for bottom-up/top-down) and confidence intervals (thin horizontal/vertical blue segments in **I**) converge to indicate that bottom-up/top-down effects may be similar at short and long durations. Exclusion of specific data points is a hazardous procedure [51, 52], however, so that this additional analysis should also be regarded as tentative.

To summarize the dependency of bottom-up/top-down effects on stimulus duration, we find some indication that the dominance of top-down information may be reduced for very short exposure, and that at such ultrashort durations the role of bottom-up information may be detectable. These observations must be viewed with caution: at an aggregate level (data collapsed across participants) the decreasing trend of top-down effects with decreasing duration is clear (inset to **Figure 6H**); the presence/absence of specific effects, however, cannot be robustly assessed at the individual observer level (**Figure 6I**).

4 Robustness tests of EEG data

Although our analysis is motivated by a fair assessment of the most relevant features displayed by the EEG, it inevitably requires arbitrary choices relating to specific parameters (e.g. filter cut-off frequencies). We therefore took additional steps to ensure that the metric we plot in **Figure 7G** is genuinely reflective of bottom-up/top-down modulations, rather than the result of arbitrary expectations imposed on noisy data [53]. First, we ran a confirmatory experiment [54,55] (see Methods). We know from the behavioural measurements that top-down effects are observed not only with natural scenes, but also with their cut-out variants (**Figure 3I**, blue symbols). We therefore expect that the top-down effects exposed by the

EEG metric in **Figure 7G** should also be measurable in response to cut-out stimuli.

4.1 Replication with cut-out stimuli

The result of applying identical analysis to additional data from EEG experiments with cut-out stimuli, shown by open symbols in **Figure 7G**, is very similar to that obtained with intact scenes (open data points fall above horizontal dashed line at p=0.03; because this is a confirmatory measurement, a one-tailed test is justified, lowering the p value to <0.02). To further validate this replication, we examined potential correlations between the combined bottom-up/top-down differential effects for cut-out stimuli (x/y values of open symbols in **Figure 7G**) and those for undistorted scenes (x/y values of solid black symbols); we found a strong correlation for occipital electrodes (see tilt of black symbols in **Figure 7I**; r value is 0.76 at p<0.002), and no significant positive correlation for central/frontal electrodes (magenta/orange data points in **Figure 7I**,**K**), further emphasizing the specificity and relevance of occipital activity.

4.2 Invariance with respect to filtering parameters and common EEG artefacts

We filtered the raw EEG with aggressive lowpass/highpass cut-off values of 20/1 Hz. These parameters were chosen to selectively target the bandwidth occupied by contralateral-ipsilateral difference waveforms [56, 57], however this choice is to some extent arbitrary. We therefore repeated our analysis with a looser bandwidth (40-0.5 Hz) to verify that the choice of cut-off parameters does not impact our results. All top-down/bottom-up effects reported in **Figure 7G-H** survive unaltered, and the estimates returned by the loose bandwidth (y axis in **Figure 7J**) are strongly correlated with those obtained with the tighter bandwidth (x axis) for occipital electrodes (black symbols in **Figure 7J**, r=0.87 at $p<10^{-12}$), but not central electrodes (p=0.36) and only mildly for frontal electrodes (r=0.36 at p<0.02). We also verified that our results do not depend on known EEG artefacts: occipital top-down effects survive artefact rejection despite the associated depletion of data mass (blue symbols in **Figure 7G** fall above horizontal dashed line at p<0.02; see Methods for details).

5 Deep networks generate proxy top-down maps

Our choice of the gain-control model is motivated by extensive electrophysiological and behavioural evidence to support this canonical computation [58, 59]. It is certainly possible to capture retuning using other schemes, but the purpose of the modelling exercise in **Figure 8C** is not tied to specific implementations: whichever circuit is favoured, the results in **Figure 8** demonstrate that top-down effects on sensitivity can be captured using a few physiologically plausible elements [60]. In other words, it is relatively straightforward to account for local processing within the probe, not only in terms of its overall characteristic, but also in relation to sensory retuning under the instruction of a putative top-down signal (blue arrows in **Figure 8C**). It is far more challenging, however, to model the instructing signal itself: it must originate from some relatively global representation of the scene, which so far we have referred to using the vague term 'top-down'.

The construction of a full-scale mechanistic model that is both physiologically plausible on the one hand, and capable of producing an adequate representation of the entire natural scene on the other hand, far exceeds our current knowledge of visual cortex [6, 61], let alone the remit of this study. We can make a first step in this direction, however, by resorting to established computer vision algorithms that, although not necessarily developed to mimic cortical processing, often share important features with neural architectures [62, 63] (see below).

5.1 Sensitivity increases gradually along top-down map

Figure 9A plots intensity on the top-down map against corresponding discrimination performance across all >1700 different probe insertion points (we pooled data across observers to obtain an average of ∼160 trials per insertion point). There is a clear correlation between the two quantities (r=0.27, p∼0). This result is expected, in that it is a different way of presenting the top-down effect discussed so far: in previous presentations (e.g. **Figure 2F**) probe insertions were classed into either rich or poor, and the associated average sensitivity values were compared (via log-ratio); **Figure 9A-C** retain the nearly continuous value on the top-down map rather than applying a binary conversion. Although the resulting correlation is partially unsurprising in light of the results reported previously, there is an interesting added benefit to examining the data in the manner plotted in **Figure 9A** (see below).

Because of the many constraints imposed on the computer algorithm that effected selection of probe locations to generate the poor/rich bottom-up/top-down structure (see Methods), the threshold between rich and poor on the top-down map classed consensus locations [64] (i.e. selected by all participants) as being rich, and all others as being poor (**Supplementary Figure 1**). The resulting classification explains the dense cluster aligned with the largest abscissa value in **Figure 9A**, the size of which is reflected by the solid green symbol. This observation prompts us to ask whether the relationship between values on the top-down map and human sensitivity is itself binary, leading to high sensitivity for consensus locations and equally low sensitivity for all other locations, or whether it presents a genuinely proportional characteristic that tracks top-down values progressively, so that the larger the top-down value, the larger the associated sensitivity. **Figure 9A** allows us to confirm the latter trend: the correlated structure remains significant (r=0.11, p<0.002) when consensus locations are excluded (i.e. by considering only data aligned with the open green symbols). This result strenghtens the connection between human sensitivity and the top-down map beyond the log-ratio analysis proposed earlier.

5.2 Deep convolutional networks (DCN) produce correlation values comparable to top-down map

As expected, there is no such correlation (r=-0.02, p=0.3) when sensitivity is similarly plotted against the bottom-up map (**Figure 9B**). As detailed in Methods, this map was generated by a basic edge detection algorithm. We now ask whether more elaborate computer vision algorithms are able to produce measurable correlations. As demonstrated in **Figure 9D**, where we present examples from three model classes, only last-generation deep networks (red) are able to generate map values that correlate significantly with human sensitivity. We focus on CRF-RNN (see full correlation plot in **Figure 9C**), a recent DCN for semantic segmentation [65] that is able to achieve a correlation value comparable to that returned by non-consensus values on the top-down map (open green symbol in **Figure 9D**; compare with right-most red symbol for CRF-RNN). To further verify that this algorithm is truly capable of capturing the top-down effects we document here, we recomputed sensitivity log-ratios in the manner adopted earlier, but with relation to the probe classification generated by the DCN rather than the top-down map derived from hand-labelling in BSD500. This analysis enables individual observer estimates, plotted in **Figure 9E**: the DCN demonstrates a clear top-down effect (red symbols fall above horizontal dashed line at p<0.01), albeit smaller in amplitude than the effect returned by the top-down map (black symbols, reproduced from Figure 2F; see confidence intervals indicated by vertical segments near y axis). For comparison, we plot the result of applying an established algorithm for image segmentation from a few years earlier [66] (preceding the deep network revolution [62]): this algorithm produces no shift in the data (blue symbols scatter around the origin), emphasizing the non-triviality of the top-down shift produced by the DCN.

5.3 DCN successfully rejects perceptually irrelevant image features

What are the features of the DCN segmentation that enable it to capture the human data in a way that other algorithms are unable to achieve? This is a complex question, but we can gain some insight by visually inspecting examples from the two algorithms. The two segmentations presented in **Figure 9E**,

corresponding to the natural scene from **Figure 1A**, demonstrate that while CRF-RNN is able to trace out the two most relevant objects in a manner consistent with human visual perception (red-tinted inset scene in **Figure 9E**), the competing algorithm presents several false alarms (blue-tinted inset scene), notably inclusive of the location (indicated by yellow circles in **Figure 9E**) that was labelled as rich on the bottom-up map but poor on the top-down map (solid red circle in Figure 1D).

6 Potential role of known phenomena and/or design flaws

6.1 Distribution of probe insertions across the visual field

We consider the following hypothetical scenario: rich probe insertions on the top-down map sample primarily the region around the fovea, while poor insertions on the top-down map fall largely within the periphery of the visual field. Further, we suppose that such differential distributions of rich versus poor locations only applies to the top-down map, and not to the bottom-up map: in relation to the latter, we suppose that rich and poor insertion points are similarly distributed across the visual field. Under this scenario, the decreased sensitivity for poor locations on the top-down map may be trivially attributed to the expected decline in sensitivity with eccentricity [67]. This interpretation is in no way connected with the notion of image understanding, potentially undermining the whole premise of our experiments and rendering the top-down effect trivially uninteresting. We can exclude this interpretation on the basis of three important observations.

First, although probe distribution is biased towards central vision, it is very similar for rich versus poor locations on both maps (**Supplementary Figure 2A**-**B**). Any small idiosyncratic difference between rich and poor distributions is similar for top-down and bottom-up maps (blue lines in **Supplementary Figure 2A**-**B**). Second, although performance did decrease with eccentricity as expected (solid/open symbols in **Supplementary Figure 2C**-**D**), the differential top-down effect was nearly unaffected by eccentricity (blue line in **Supplementary Figure 2D**). In other words, even when the analysis is restricted to a specific subregion of the visual field and whichever that region may be, it is still the case that the top-down effect is present (and unchanged in magnitude), while the bottom-up effect is absent (blue line in **Supplementary Figure 2C**). Third and most importantly, if the top-down effect is merely the result of differential probe distribution, it should survive the 'line' manipulation (**Figure 3H**): in the experiments with line-manipulated images, probe distribution across the visual field is statistically identical to that used with intact natural scenes. Contrary to this expectation, the top-down effect was absent with line-manipulated images (red symbols in **Figure 3I**), providing conclusive evidence against the proposed artefactual interpretation of top-down effects.

6.2 Second-order texture cues

Research on eye-movement control has demonstrated, alongside the general importance of top-down factors [68], the need to consider not only first-order image attributes when evaluating the low-level content of natural scenes [69], but also second-order cues [70]. The bottom-up map defined in **Figure 1C** (main text) relies on first-order energy alone, because edge detection as supported by differential operators [71] (and many other similar edge-detection algorithms) is luminance-based. It is therefore conceivable that the rich/poor categorizaton afforded by the bottom-up map may completely miss second-order image cues, such as edges defined by spatial frequency inhomogeneities [70], and that it is these low-level cues that drive top-down effects. We can rule out this interpretation on the basis of two important observations. First, the specifics of how the bottom-up map is generated are unimportant to our conclusions, as long as they retain the notion of low-level representation. For example, we show that human sensitivity is uncorrelated with image content when this is coded along the bottom-up map generated by the luminance operator (black symbol in **Figure 9D**), as well as one generated by visual saliency algorithms that do incorporate second-order cues such as the Itti-Koch operator [72] (orange symbols). Second and most importantly, if the top-down effect is driven by second-order cues, it should be entirely eliminated by the cut-out manipulation (**Figure 3G**), because cut-out images destroy all second-order structure that may have been present within the original natural scenes (see paragraph

below for further elaboration on this point). Contrary to this expectation, the top-down effect was clearly measurable with cut-out images (blue symbols in **Figure 3I**), providing conclusive evidence against the proposed artefactual interpretation.

It is useful to consider in greater detail exactly *why* cut-out images eliminate any potential connection between second-order cues on the one hand, and labelling across bottom-up/top-down maps on the other hand. This outcome critically depends on a specific design feature of the probe insertion algorithm, namely that no probe insertion point could occur at 0 values on either top-down or bottom-up maps (see Methods section within main text): even for the case of 'poor' labels, the probe insertion point was restricted to a minimal threshold value on the respective map. For example, a probe that was labelled 'poor' on the top-down map was nevertheless associated with a minimal non-zero value on the top-down map. In turn, this means that the associated boundary would be retained by the cut-out manipulation (as well as the 'line' variant, **Figure 3H**).

Suppose that we now measure low-level content from cut-out images in the form of a 'surrogate' low-level map; there is no low-level information in the cut-out images other than luminance-defined edges, so first-order cues are sufficient to capture all low-level content within cut-out images. All insertion points (whether originally labelled 'poor' or 'rich' on the top-down map) will be associated with a visible boundary on said 'surrogate' map, and the map value associated with each boundary will be randomly assigned by virtue of the random luminance assignment given to different regions of cut-out images. The end result is that the 'surrogate' map, and therefore the low-level content in the cut-out image, is completely disconnected from labelling on the top-down map. Whatever role second-order cues may play in the experiments, whether in relation to the manner in which they were exploited by participants in BSD500 for boundary annotation or in relation to their potential contribution to the top-down effects we measure here, it is inapplicable in the case of cut-out images: in order for cut-out images to generate a top-down effect, their perceptual representation must share some similarities with the original scenes, and these similarities must derive from image features that are not captured by low-level content.

A similar conclusion is reached by considering 'line' images, which present the same boundaries contained within cut-out images. In both cases, all image boundaries from the original scene are converted into luminance-defined objects of equal magnitude: exactly equal in the case of 'line' images, statistically equal (on average across random assignments) in the case of 'cut-out' images. Any potential role played by second-order cues in natural scenes must carry over to both manipulations, yet top-down effects are only observed for one ('cut-out') and not the other ('line'), ruling out any low-level role for second-order cues.

It is important to clarify that our experiments do *not* exclude a role for second-order cues in assisting the perceptual reconstruction of object boundaries from natural scenes; nor, for that matter, do they exclude such a role for first-order cues: clearly, the perceptual representation of the scene must come from the image, as there is nothing else in the stimulus that provides observers with information about the scene to be interpreted. The issue is one of separating incidental from critical factors. Low-level image cues, whether of the first- or second-order kind, are incidental in the sense that, although they provide image definition that is exploited to obtain a perceptual representation of the layout of the scene, such representation can be achieved by relying on a variety of image features, and ultimately it is unimportant which specific low-level cues underlie the representation: only the latter is critical to observe the top-down effects measured in this study, *not* the low-level cues that were exploited to obtain the representation in the first place.

6.3 Spatial uncertainty

Previous literature has recognized that spatial uncertainty can often account for a wide range of phenomena [73], many of which may not at first appear connected with this notion [74]. We must therefore ask whether the top-down effects may be explained by uncertainty reduction: is it conceivable that rich locations on the top-down map may be associated with less uncertainty than poor locations, resulting in enhanced sensitivity? Several considerations lead us to exclude this interpretation. First, the precue/postcue manipulation involves a very substantial reduction of spatial uncertainty, in turn associated

with a 2-fold improvement in performance (magenta symbols in **Figure 2E**). This otherwise sizeable manipulation had no impact on top-down effects (**Figure 2G**), strongly indicating that the two factors are orthogonal. Second, spatial uncertainty is larger in the peripheral visual field [75]; if this phenomenon played a role in determining top-down effects, such effects should be more pronounced in the periphery than in the fovea. Contrary to this prediction, we found that top-down effects were at least as pronounced, if not more so, in the central visual field as opposed to the periphery (**Supplementary Figure 2D**). Third, prior work has demonstrated that spatial attention is not associated with orientation retuning across a wide range of stimulus/task specifications [76–78], including some not dissimilar than the probe design adopted here [24]. In contrast, the top-down effects we document in this study are associated with sizeable retuning (**Figure 8C**; blue symbols in **Figure 8B**). In conclusion, the notion of spatial uncertainty cannot account for the primary effects reported in this study.

6.4 Crowding

Crowding is an additional prominent factor known to impact local discrimination [79, 80]. We may hypothesize that, for whatever reason, crowding may be less pronounced at rich locations within the top-down map. There is no doubt that crowding was operational during the experiments detailed here, due to the mere presence of complex visual elements immediately surrounding the probe region. It is extremely unlikely, however, that its strength varied systematically in such a manner as to generate the top-down effects reported in this study. First, crowding is primarily (if not exclusively) a peripheral phenomenon in the intact visual system [81], whereas top-down effects show virtually no dependence on eccentricity (**Supplementary Figure 2D**). Second, it has been claimed that crowding is intimately related to the notion of summary statistic [82], and therefore primarily connected with impaired discrimination of second-order inter-feature relationships [83] that may be lost when highly structured properties of image patches are reduced to summary statistical descriptions [84]. In our experiments, discrimination relied on the extraction of a single basic feature (orientation), the statistics of which would be largely retained by minimal summary accounts. Third, crowding is greatly affected by the introduction of asynchronous timing between probe and crowders [85]; this effect depends on presentation order, with stronger crowding for probes preceding crowders [86]. If crowding played a substantial role in shaping top-down effects, they should be reduced when probe and natural scene are asynchronous, and more importantly this reduction should be asymmetrical with respect to probe-crowder temporal order. Contrary to this prediction, the top-down effects we observe with zooming stimuli are of comparable magnitude to those obtained with synchronous elements (compare red/magenta bars next to y axis in **Figure 5F** (main text) with red/black bars next to y axis in **Figure 2F**) and do not depend on zooming direction (compare blue bar with light-blue bar next to y axis in **Figure 5G**). In conclusion, crowding cannot account for the top-down effects reported in this study.

6.5 Flanker effects

In the same way that crowding must have been active during the experiments (but not contributing differential effects), so must have been flanker facilitation/inhibition [87]. Various elements of our dataset rule out this well-known phenomenon as a potential explanation for top-down effects. Some of those elements have been presented above; here we offer additional observations. First, under the expectation that the region flanking the probe should impact sensitivity, the largest effects must be expected for the bottom-up, not the top-down, manipulation, because the latter involves far larger changes in local characteristics than the former (see **Figure 2A**-**D** in main text). Furthermore, if top-down effects are caused by the flanking region, we expect those effects to disappear when said region is removed, contrary to what we observed in the gap experiments (**Figure 6A**-**G** in main text). We can therefore exclude that flanking facilitation/inhibition is the source of the top-down effects reported here.

6.6 Eye movements

Eye-movement scanning of natural scenes often presents idiosyncratic characteristics [68, 88], and it has been claimed that several low-level image features may contribute, some of which (e.g. second-order

texture cues) may be overlooked by edge-detection algorithms [69, 70]. Our experiments with cut-out scenes, among others, rule out the possibility that top-down effects may depend on such features to any substantial extent (see preceding section on 'Second-order texture cues'), however eye-movement strategies may play a role. It is conceivable, for example, that eye movements may differ when probes are inserted at rich versus poor locations on the top-down map, differentially affecting sensitivity. This interpretation, however, is ruled out by the spatial cueing experiments. We emphasize that observers were clearly instructed to maintain fixation at all times and stimulus duration was below typical eye-movement rate ($\frac{1}{3}$Hz), thus minimizing any potential role of eye movements. If however they nonetheless moved their eyes, we expect that their eye-movement strategy should be subtantially different on precue trials, when they had prior information as to where the probe would appear and may therefore be tempted to foveate that location, as opposed to postcue trials. If top-down effects reflect eye-movement strategy, they should differ between precue and postcue trials, contrary to our observations (**Figure 2G**).

A related argument applies to the zooming stimuli: zooming *direction* is expected to impact eye-movement strategy due to its differing focus-in/focus-out characteristics, and it may be reasonable to expect that zooming *duration* would also play a role. In particular, it is expected that eye-movements would be drawn to the probe much more quickly for the zooming-out stimulus due to earlier knowledge of probe location, while they would be guided to probe location more gradually under zooming-in condition; further, it is expected that the associated difference in eye-movement strategy would be more pronounced at longer durations of 300 ms, comparable with the frequency of eye movements, than at 100 ms, a period too short to allow for such differences to emerge. Contrary to these expectations, neither direction nor duration had any impact on top-down effects (**Figure 5F**-**G**). Combined with the observations in the previous paragraph, we conclude that eye movements are not the source of the top-down effects reported in this study.

6.7 Criterion shifts

The primary behavioural metric of interest in this study is visual sensitivity in the form of d′, an established concept from signal detection theory [89] (SDT). It is well-known that, under some conditions, d′ may be confounded with criterion changes [90], making 2-alternative-forced-choice (2AFC) designs preferable to yes-no single-interval protocols because, under the former design, criterion effects are minimized/eliminated [89]. Unfortunately, it was not possible to design the experiments described here in conformity with 2AFC protocols. In our previous work with natural scenes [26, 91], we adopted spatial 2AFC designs where observers saw two identical scenes symmetrically presented on opposite sides of fixation. Although this design enables bias minimization [89], it involves several shortcomings: 1) the stimulus is very unnatural, because we never experience natural scenes as duplicated on opposite sides of the fovea; 2) probe insertions can only occur peripherally, precluding the kind of eccentricity analysis afforded by **Supplementary Figure 2** and associated conclusions (see above); 3) most importantly, the 2AFC spatial design would make it impossible to peform the lateralized analysis that is critical to the EEG experiment (**Figure 7**) because the presence of two probes on opposite sides of fixation would preclude attribution of specific EEG modulations to the spatial location of individual probes. Similar criticism applies to temporal 2AFC variants: besides implicating memory as a potential confounding factor, these protocols are particularly problematic in relation to the EEG experiments because the VEP associated with the second interval would inevitably present superimposed activity from the first interval, making it difficult (if not impossible) to correctly interpret relevant patterns [57]. We therefore opted for a yes-no single-interval paradigm, which results in more natural stimulation and viable task conditions. To minimize any potential role for criterion changes, we mixed the primary conditions of interest (bottom-up/top-down poor/rich) within the same block. We further confirm below that the d′ changes we measure in association with bottom-up/top-down effects conclusively reflect true sensitivity changes; we do measure, however, concomitant criterion effects, which we document below.

Within the context of the equal-variance SDT model, d′ and criterion c are computed as $\Phi^{-1}(p_{\text{hit}}) - \Phi^{-1}(p_{\text{fa}})$ and $-0.5 \times [\Phi^{-1}(p_{\text{hit}}) + \Phi^{-1}(p_{\text{fa}})]$, where Φ^{-1} is the inverse of the normal cumulative distribution function, p_{hit} is the probability of a hit response, and p_{fa} the probability of a false alarm [89]. In our

experiments these two quantities are clearly correlated (**Supplementary Figure 3B**, r=0.75, $p<10^{-6}$), so that several top-down effects reported in this study with respect to d′ can also be demonstrated with respect to c. This may raise suspicions about the origin of the d′ changes, and in particular whether they may not result from criterion changes for an underlying mechanism that does not conform to the assumed equal-variance model. We can rule out this possibility based on several observations.

We first notice that criterion shifts are not only commonly measured in concomitance with sensitivity changes [92–95], but are also expected from plausible theoretical considerations [94, 96]. In light of existing literature we conclude that their absence, rather than their presence (as we observe here), would raise legitimate suspicions. Furthermore, and in line with previous work [94], the criterion effects we measure are nearly one order of magnitude smaller than corresponding sensitivity effects. The most transparent way of appreciating this fact is to plot our dataset as receiver operating characteristics [90] (ROC), as we do in **Supplementary Figure 3A**. This approach retains close proximity with the raw data: there are no assumptions underlying this manner of summarizing behaviour, in that ROC plots simply report direct estimates of the probability associated with specific stimulus-response outcomes. More specifically, for a yes-no single-interval protocol we can summarize behaviour using only p_{fa} and p_{hit} (see above), plotted on x and y axes respectively in **Supplementary Figure 3A**. Two basic conclusions can be easily arrived at from evaluating the data in this manner.

First, observers were consistently biased in the direction of adopting a conservative criterion: they were substantially more likely to report that the probe was incongruent rather than congruent (data points fall below diagonal magenta line corresponding to no response bias or c=0). The presence of such bias makes it imperative that potential criterion shifts are analyzed thoroughly [97], as we do here. Second, the poor→rich transition on the top-down map indicated by the red→green cluster separation (see inset to **Supplementary Figure 3A**) is much more pronounced along the direction corresponding to a sensitivity change (up to the left, see magenta arrow within main panel) than along the direction corresponding to a criterion change (down to the left, see magenta arrow within inset). Readers familiar with ROC plots can immediately gauge from the data scatter that the transition effected by the red→green clusters is not compatible with any iso-sensitivity fit from plausible unequal-variance SDT models [89, 90]: it is bound to reflect a substantial change in sensitivity. This sensitivity change may be associated with a small criterion effect, as indicated by our measurements and as expected on theoretical grounds [94, 96], but it is certainly not artefactually generated by the criterion change.

To further appreciate the difference in magnitude between changes in d′ and c shifts, we plot best-fit traces from an equal-variance SDT model to the different data clusters. Solid traces in **Supplementary Figure 3A** correspond to different sensitivity values (isosensitivity curves); they demonstrate a clear transition along the top-down map (red→green) with no transition along the bottom-up map (gray→black). This is the top-down effect on sensitivity which we have documented extensively in the main text via log-ratios (e.g. **Figure 2F**). Dashed traces in the inset to **A** correspond to different criterion values (isocriterion curves); they demonstrate a concomitant transition along the top-down map (red→green), however its magnitude is substantially smaller than the corresponding transition for sensitivity. A more direct and quantitative approach for evaluating this magnitude difference is to gather rich/poor log-ratios for both d′ and c across all experimental conditions that showed a top-down effect with no bottom-up effect, and plot them against each other in **Supplementary Figure 3C**. For d′ (y axis), the 95% confidence interval on the top-down map (green vertical segment near right y axis) is 0.36-0.54; for c it is 0.04-0.1 (green horizontal segment near top x axis), i.e. nearly ×7 times smaller. Further, there is no correlation between d′ and c when recast as top-down rich/poor log-ratios (r=-0.02, p=0.89); the lack of correlation for differential effects re-enforces the notion that, although the two processes correlate on absolute scales (**Supplementary Figure 3B**), they are independently modulated by top-down control.

To summarize this section, we find small and largely expected [92–96] criterion shifts associated with the reported sensitivity transitions. The presence of these effects is of no concern for our d′-based analysis, particularly in the form of log-ratios (**Supplementary Figure 3C**).

References

[1] M. Riesenhuber and T. Poggio. Hierarchical models of object recognition in cortex. *Nat. Neurosci.*, 2(11):1019–1025, Nov 1999.

[2] D. C. Marr. *Vision: A Computational Investigation into the Human Representation and Processing of Visual Information*. New York: Freeman, 1982.

[3] J. Kubilius, J. Wagemans, and H. P. Op de Beeck. A conceptual framework of computations in mid-level vision. *Front Comput Neurosci*, 8:158, 2014.

[4] J. W. Peirce. Understanding mid-level representations in visual processing. *J Vis*, 15(7):5, 2015.

[5] S. Ullman. *High-level vision*. Cambridge, MA: MIT Press, 1996.

[6] D. D. Cox. Do we understand high-level vision? *Curr. Opin. Neurobiol.*, 25:187–193, Apr 2014.

[7] R. Shapley. Early vision is early in time. *Neuron*, 56(5):755–756, Dec 2007.

[8] P. R. Roelfsema, M. Tolboom, and P. S. Khayat. Different processing phases for features, figures, and selective attention in the primary visual cortex. *Neuron*, 56(5):785–792, Dec 2007.

[9] R. K. Yin. Looking at upside-down faces. *J Exp Psychol*, 81:141–145, Nov 1969.

[10] P. Thompson and M. Thatcher. Margaret Thatcher: a new illusion. *Perception*, 9:483–484, 1980.

[11] T. Valentine. Upside-down faces: a review of the effect of inversion upon face recognition. *Br J Psychol*, 79 (Pt 4):471–491, Nov 1988.

[12] T. A. Kelley, M. M. Chun, and K. P. Chua. Effects of scene inversion on change detection of targets matched for visual salience. *J Vis*, 3(1):1–5, 2003.

[13] D. B. Walther, E. Caddigan, L. Fei-Fei, and D. M. Beck. Natural scene categories revealed in distributed patterns of activity in the human brain. *J. Neurosci.*, 29(34):10573–10581, Aug 2009.

[14] C. M. Gaspar, P. J. Bennett, and A. B. Sekuler. The effects of face inversion and contrast-reversal on efficiency and internal noise. *Vision Res.*, 48:1084–1095, Mar 2008.

[15] M. A. Cohen, G. A. Alvarez, and K. Nakayama. Natural-scene perception requires attention. *Psychol Sci*, 22(9):1165–1172, Sep 2011.

[16] B. M. 't Hart, H. C. Schmidt, I. Klein-Harmeyer, and W. Einhauser. Attention in natural scenes: contrast affects rapid visual processing and fixations alike. *Philos. Trans. R. Soc. Lond., B, Biol. Sci.*, 368(1628):20130067, Oct 2013.

[17] I. I. Groen, S. Ghebreab, V. A. Lamme, and H. S. Scholte. The time course of natural scene perception with reduced attention. *J. Neurophysiol.*, 115(2):931–946, Feb 2016.

[18] F. F. Li, R. VanRullen, C. Koch, and P. Perona. Rapid natural scene categorization in the near absence of attention. *Proc. Natl. Acad. Sci. U.S.A.*, 99:9596–9601, Jul 2002.

[19] G. A. Rousselet, M. Fabre-Thorpe, and S. J. Thorpe. Parallel processing in high-level categorization of natural images. *Nat. Neurosci.*, 5:629–630, Jul 2002.

[20] K. K. Evans and A. Treisman. Perception of objects in natural scenes: is it really attention free? *J Exp Psychol Hum Percept Perform*, 31(6):1476–1492, Dec 2005.

[21] W. Einhauser, T. N. Mundhenk, P. Baldi, C. Koch, and L. Itti. A bottom-up model of spatial attention predicts human error patterns in rapid scene recognition. *J Vis*, 7(10):1–13, 2007.

[22] M. V. Peelen, L. Fei-Fei, and S. Kastner. Neural mechanisms of rapid natural scene categorization in human visual cortex. *Nature*, 460(7251):94–97, Jul 2009.

[23] T. Liu, F. Pestilli, and M. Carrasco. Transient attention enhances perceptual performance and FMRI response in human visual cortex. *Neuron*, 45(3):469–477, Feb 2005.

[24] A. E. Paltoglou and P. Neri. Attentional control of sensory tuning in human visual perception. *J Neurophysiol*, 107:1260–1274, 2012.

[25] I. Biederman. Perceiving real-world scenes. *Science*, 177:77–80, Jul 1972.

[26] P. Neri. Semantic control of feature extraction from natural scenes. *J. Neurosci.*, 34(6):2374–2388, Feb 2014.

[27] R. L. DeValois and K. K. DeValois. *Spatial Vision*. New York: Oxford University Press, 1988.

[28] J. A. Mazer, W. E. Vinje, J. McDermott, P. H. Schiller, and J. L. Gallant. Spatial frequency and orientation tuning dynamics in area V1. *PNAS*, 99:1645–1650, Feb 2002.

[29] J. H. Elder. Are Edges Incomplete? *IJCV*, 34(2):97–122, Aug 1999.

[30] O Henaff, N Rabinowitz, J Ballé, and E P Simoncelli. The local low-dimensionality of natural images. In *Int'l. Conf. on Learning Representations (ICLR2015)*, San Diego, CA, May 2015.

[31] J. Hegde. Time course of visual perception: coarse-to-fine processing and beyond. *Prog. Neurobiol.*, 84(4):405–439, Apr 2008.

[32] P. Neri. Coarse to fine dynamics of monocular and binocular processing in human pattern vision. *Proc. Natl. Acad. Sci. U.S.A.*, 108:10726–10731, Jun 2011.

[33] V. Goffaux, J. Peters, J. Haubrechts, C. Schiltz, B. Jansma, and R. Goebel. From coarse to fine? Spatial and temporal dynamics of cortical face processing. *Cereb. Cortex*, 21(2):467–476, Feb 2011.

[34] A. De Cesarei and G. R. Loftus. Global and local vision in natural scene identification. *Psychon Bull Rev*, 18(5):840–847, Oct 2011.

[35] L. N. Piotrowski and F. W. Campbell. A demonstration of the visual importance and flexibility of spatial-frequency amplitude and phase. *Perception*, 11:337–346, 1982.

[36] M. G. Thomson, D. H. Foster, and R. J. Summers. Human sensitivity to phase perturbations in natural images: a statistical framework. *Perception*, 29(9):1057–1069, 2000.
[37] G. Felsen, J. Touryan, F. Han, and Y. Dan. Cortical sensitivity to visual features in natural scenes. *PLoS Biol.*, 3:e342, Oct 2005.
[38] J. M. Hupe, A. C. James, B. R. Payne, S. G. Lomber, P. Girard, and J. Bullier. Cortical feedback improves discrimination between figure and background by V1, V2 and V3 neurons. *Nature*, 394:784–787, Aug 1998.
[39] J. W. Rieger, N. Kochy, F. Schalk, M. Gruschow, and H. J. Heinze. Speed limits: orientation and semantic context interactions constrain natural scene discrimination dynamics. *J Exp Psychol Hum Percept Perform*, 34(1):56–76, Feb 2008.
[40] J. Bullier. Feedback connections and conscious vision. *Trends Cogn. Sci. (Regul. Ed.)*, 5(9):369–370, Sep 2001.
[41] M. Bar. Visual objects in context. *Nat. Rev. Neurosci.*, 5(8):617–629, Aug 2004.
[42] O. R. Joubert, G. A. Rousselet, D. Fize, and M. Fabre-Thorpe. Processing scene context: fast categorization and object interference. *Vision Res.*, 47(26):3286–3297, Dec 2007.
[43] C. W. Eriksen. The use of a visual mask may seriously confound your experiment. *Percept Psychophys*, 28(1):89–92, Jul 1980.
[44] B. C. Hansen and R. F. Hess. On the effectiveness of noise masks: naturalistic vs. un-naturalistic image statistics. *Vision Res.*, 60:101–113, May 2012.
[45] T. U. Otto, H. Öğmen, and M. H. Herzog. The flight path of the phoenix–the visible trace of invisible elements in human vision. *J Vis*, 6(10):1079–1086, Sep 2006.
[46] R. Van Rullen. Four common conceptual fallacies in mapping the time course of recognition. *Front Psychol*, 2:365, 2011.
[47] F. Hermens, M. H. Herzog, and G. Francis. Combining simultaneous with temporal masking. *J Exp Psychol Hum Percept Perform*, 35(4):977–988, Aug 2009.
[48] M. H. Herzog, T. Kammer, and F. Scharnowski. Time Slices: What Is the Duration of a Percept? *PLoS Biol.*, 14(4):e1002433, Apr 2016.
[49] C. R. Pernet, R. Wilcox, and G. A. Rousselet. Robust correlation analyses: false positive and power validation using a new open source matlab toolbox. *Front Psychol*, 3:606, 2012.
[50] K. R. Gegenfurtner and J. Rieger. Sensory and cognitive contributions of color to the recognition of natural scenes. *Curr. Biol.*, 10(13):805–808, Jun 2000.
[51] F. E. Grubbs. Procedures for detecting outlying observations in samples. *Technometrics*, 11(1):1–21, 1969.
[52] J. N. Miller. Tutorial review-outliers in experimental data and their treatment. *Analyst*, 118:455–461, 1993.
[53] E. Vul, C. Harris, P. Winkielman, and H. Pashler. Puzzlingly High Correlations in fMRI Studies of Emotion, Personality, and Social Cognition. *Perspect Psychol Sci*, 4(3):274–290, May 2009.
[54] J. P. Ioannidis. Why most published research findings are false. *PLoS Med.*, 2(8):e124, Aug 2005.
[55] G. Cumming. The new statistics: why and how. *Psychol Sci*, 25(1):7–29, Jan 2014.
[56] S. J. Luck and S. A. Hillyard. Spatial filtering during visual search: evidence from human electrophysiology. *J Exp Psychol Hum Percept Perform*, 20(5):1000–1014, Oct 1994.
[57] S. J. Luck. *An Introduction to the Event-Related Potential Technique*. MIT Press, 2005.
[58] M. Carandini and D. J. Heeger. Normalization as a canonical neural computation. *Nat. Rev. Neurosci.*, 13:51–62, 2011.
[59] N. C. Rabinowitz, B. D. Willmore, J. W. Schnupp, and A. J. King. Contrast gain control in auditory cortex. *Neuron*, 70(6):1178–1191, Jun 2011.
[60] P. Neri. The elementary operations of human vision are not reducible to template matching. *PLoS Comput. Biol.*, 11(11):e1004499, 2015.
[61] J. J. DiCarlo, D. Zoccolan, and N. C. Rust. How does the brain solve visual object recognition? *Neuron*, 73(3):415–434, Feb 2012.
[62] N. Kriegeskorte. Deep Neural Networks: A New Framework for Modeling Biological Vision and Brain Information Processing. *Annual Review of Vision Science*, 1:417–446, 2015.
[63] D. L. Yamins and J. J. DiCarlo. Using goal-driven deep learning models to understand sensory cortex. *Nat. Neurosci.*, 19(3):356–365, Feb 2016.
[64] Xiaodi Hou, Alan L. Yuille, and Christof Koch. Boundary detection benchmarking: Beyond f-measures. In *CVPR*, pages 2123–2130. IEEE Computer Society, 2013.
[65] Shuai Zheng, Sadeep Jayasumana, Bernardino Romera-Paredes, Vibhav Vineet, Zhizhong Su, Dalong Du, Chang Huang, and Philip Torr. Conditional random fields as recurrent neural networks. In *International Conference on Computer Vision (ICCV)*, 2015.
[66] Pablo Arbelaez, Michael Maire, Charless Fowlkes, and Jitendra Malik. Contour detection and hierarchical image segmentation. *IEEE Trans Patt Anal Mach Intell*, 33(5):898–916, May 2011.
[67] H. Strasburger, I. Rentschler, and M. Juttner. Peripheral vision and pattern recognition: a review. *J Vis*, 11(5):13, 2011.
[68] M. Nyström and K. Holmqvist. Semantic Override of Low-level Features in Image Viewing Both Initially and Overall. *J Eye Movement Research*, 2(2):1–11, 2008.
[69] W. Einhauser and P. Konig. Does luminance-contrast contribute to a saliency map for overt visual attention? *Eur.*

J. Neurosci., 17(5):1089–1097, Mar 2003.

[70] D. J. Parkhurst and E. Niebur. Texture contrast attracts overt visual attention in natural scenes. *Eur. J. Neurosci.*, 19(3):783–789, Feb 2004.

[71] H. Farid and E. P. Simoncelli. Differentiation of discrete multidimensional signals. *IEEE Trans Image Process*, 13(4):496–508, Apr 2004.

[72] L. Itti and C. Koch. Computational modelling of visual attention. *Nat. Rev. Neurosci.*, 2(3):194–203, Mar 2001.

[73] D. G. Pelli. Uncertainty explains many aspects of visual contrast detection and discrimination. *J Opt Soc Am A*, 2:1508–1532, Sep 1985.

[74] C. A. Perez, T. E. Cohn, L. E. Medina, and J. R. Donoso. Coincidence-enhanced stochastic resonance: experimental evidence challenges the psychophysical theory behind stochastic resonance. *Neurosci. Lett.*, 424:31–35, Aug 2007.

[75] R. F. Hess and D. Field. Is the increased spatial uncertainty in the normal periphery due to spatial undersampling or uncalibrated disarray? *Vision Res.*, 33(18):2663–2670, Dec 1993.

[76] P. Neri. Attentional effects on sensory tuning for single-feature detection and double-feature conjunction. *Vision Res.*, 44:3053–3064, Dec 2004.

[77] S. Baldassi and P. Verghese. Attention to locations and features: different top-down modulation of detector weights. *J Vis*, 5:556–570, 2005.

[78] S. Ling, J. F. Jehee, and F. Pestilli. A review of the mechanisms by which attentional feedback shapes visual selectivity. *Brain Struct Funct*, 220(3):1237–1250, 2015.

[79] D. G. Pelli and K. A. Tillman. The uncrowded window of object recognition. *Nat. Neurosci.*, 11(10):1129–1135, Oct 2008.

[80] D. M. Levi. Visual crowding. *Curr. Biol.*, 21:R678–679, Sep 2011.

[81] D. G. Pelli, M. Palomares, and N. J. Majaj. Crowding is unlike ordinary masking: distinguishing feature integration from detection. *J Vis*, 4:1136–1169, Dec 2004.

[82] B. Balas, L. Nakano, and R. Rosenholtz. A summary-statistic representation in peripheral vision explains visual crowding. *J Vis*, 9(12):1–18, 2009.

[83] P. Neri and D. M. Levi. Spatial resolution for feature binding is impaired in peripheral and amblyopic vision. *J. Neurophysiol.*, 96:142–153, Jul 2006.

[84] J. H. McDermott, M. Schemitsch, and E. P. Simoncelli. Summary statistics in auditory perception. *Nat. Neurosci.*, 16(4):493–498, Apr 2013.

[85] A. Huckauf and D. Heller. On the relations between crowding and visual masking. *Percept Psychophys*, 66(4):584–595, May 2004.

[86] S. T. Chung. Spatio-temporal properties of letter crowding. *J Vis*, 16(6):8, 2016.

[87] C. Yu and D. M. Levi. Surround modulation in human vision unmasked by masking experiments. *Nat. Neurosci.*, 3:724–728, Jul 2000.

[88] S. C. Mack and M. P. Eckstein. Object co-occurrence serves as a contextual cue to guide and facilitate visual search in a natural viewing environment. *J Vis*, 11(9):1–16, 2011.

[89] D. M. Green and J. A. Swets. *Signal Detection Theory and Psychophysics*. New York: Wiley, 1966.

[90] J. A. Swets. Form of empirical ROCs in discrimination and diagnostic tasks: implications for theory and measurement of performance. *Psychol Bull*, 99(2):181–198, Mar 1986.

[91] P. Neri. Global properties of natural scenes shape local properties of human edge detectors. *Front Psychol*, 2:172, 2011.

[92] H. J. Muller and J. M. Findlay. Sensitivity and criterion effects in the spatial cuing of visual attention. *Percept Psychophys*, 42(4):383–399, Oct 1987.

[93] H. L. Hawkins, S. A. Hillyard, S. J. Luck, M. Mouloua, C. J. Downing, and D. P. Woodward. Visual attention modulates signal detectability. *J Exp Psychol Hum Percept Perform*, 16(4):802–811, Nov 1990.

[94] P. Martini and V. Maljkovic. Short-term memory for pictures seen once or twice. *Vision Res.*, 49(13):1657–1667, Jun 2009.

[95] K. C. Aberg and M. H. Herzog. Different types of feedback change decision criterion and sensitivity differently in perceptual learning. *J Vis*, 12(3), 2012.

[96] M. Glanzer, A. Hilford, and L. T. Maloney. Likelihood ratio decisions in memory: three implied regularities. *Psychon Bull Rev*, 16(3):431–455, Jun 2009.

[97] A. Hilford, L. T. Maloney, M. Glanzer, and K. Kim. Three regularities of recognition memory: the role of bias. *Psychon Bull Rev*, 22(6):1646–1664, Dec 2015.
