# Supplementary figures and images for: Object segmentation controls image reconstruction from natural scenes
Source: PLoS Biol. 2017 Aug 21;15(8):e1002611. doi: 10.1371/journal.pbio.1002611 (PMC5565198; doi:10.1371/journal.pbio.1002611)

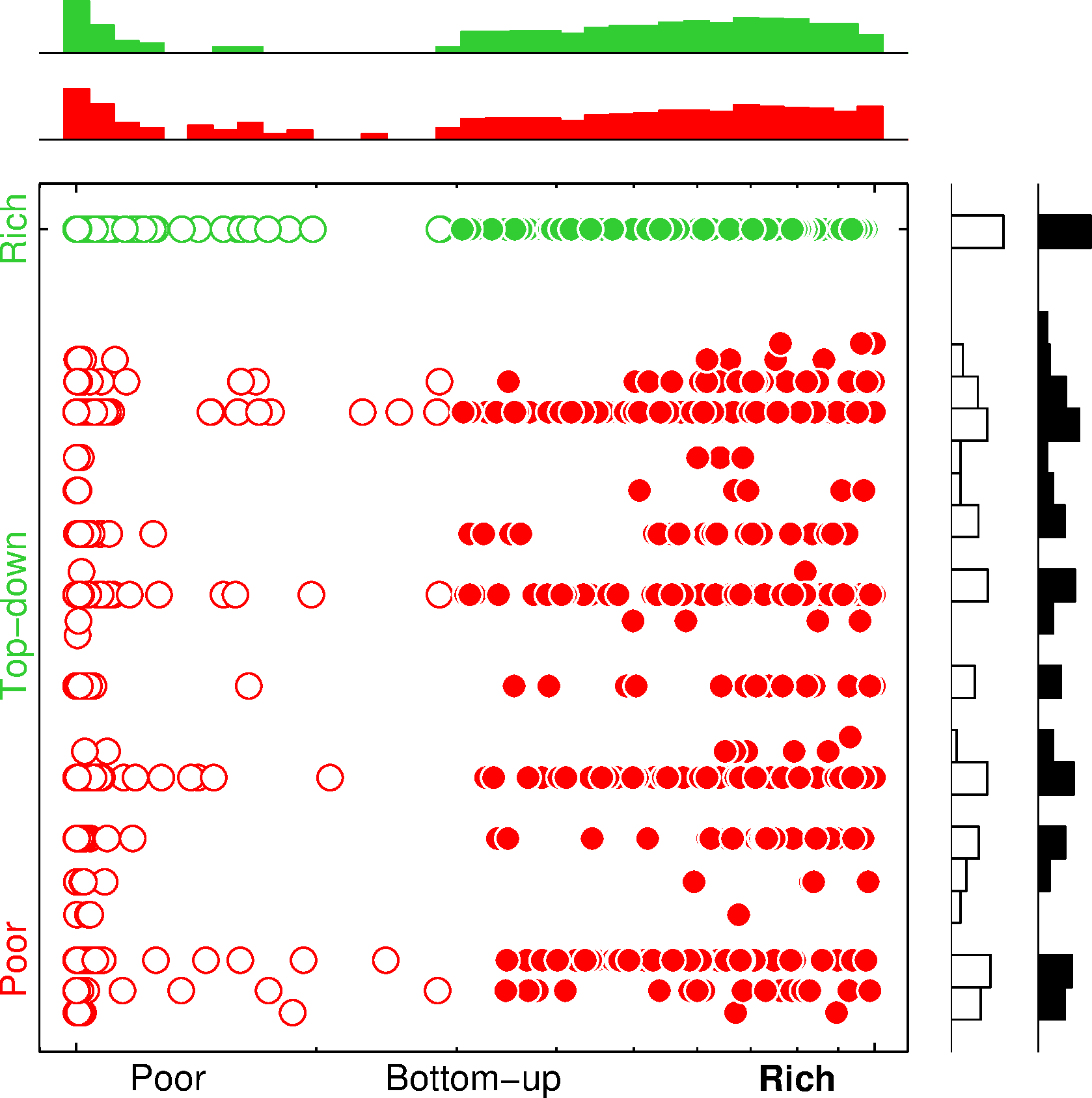

Supplement: S1 Fig — Value on top-down map (y axis) is plotted against value on bottom-up map (x axis) across all probe insertions (1 symbol per insertion); open/solid indicates poor/rich on bottom-up map, red/green indicates poor/rich on top-down map. Marginal distributions along the bottom-up map (top histograms) are virtually identical for poor/rich locations on top-down map (red/green solid histograms); similarly, marginal distributions along the top-down map (right histograms) are indistinguishable for poor/rich locations on bottom-up map (open/solid histograms). Standard correlation tests are not applicable because this dataset is not normally distributed (Henze-Zirkler test) and it is heteroscedastic (test based on conditional variances). (TIF) [file pbio.1002611.s002.tif]

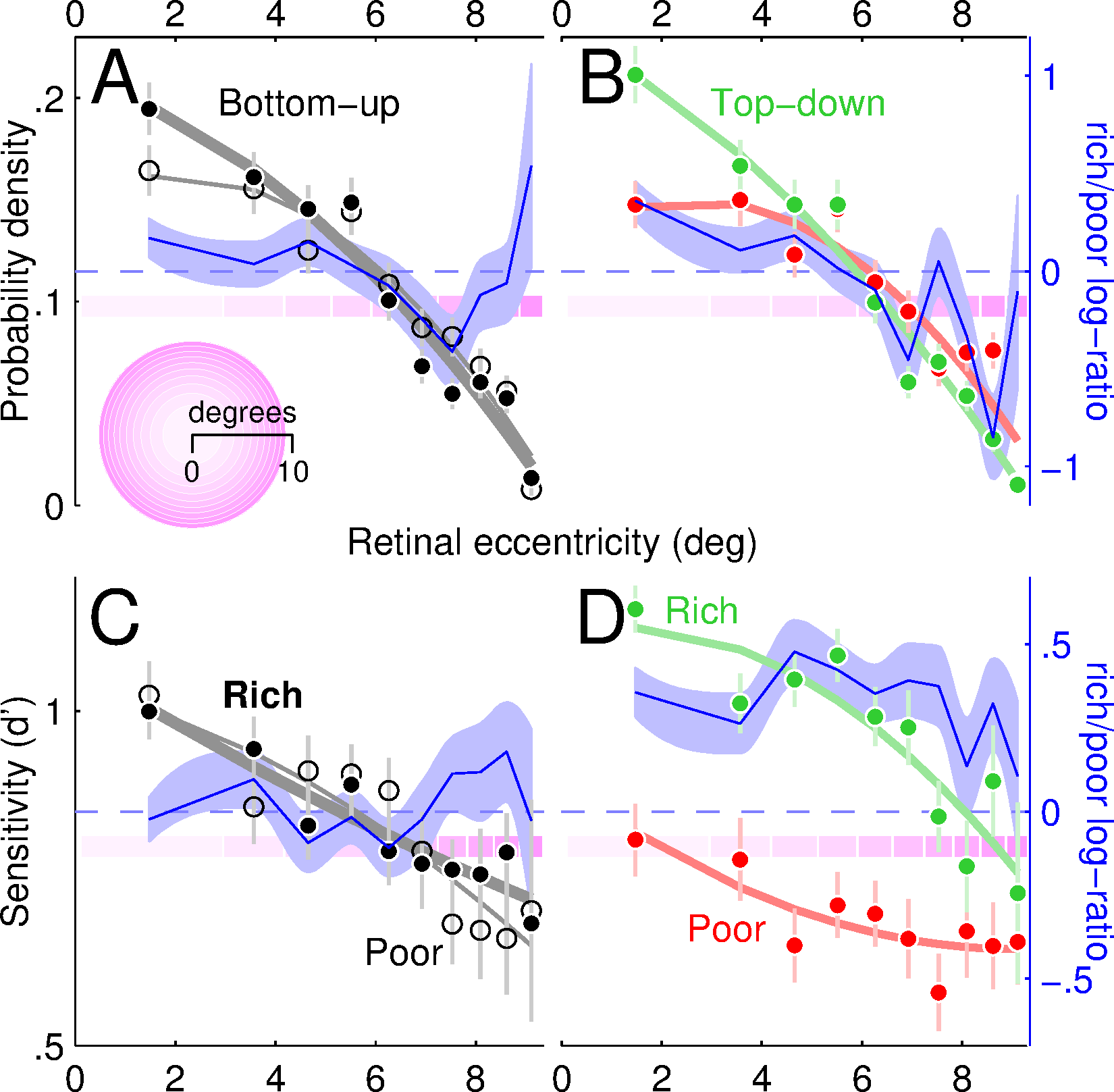

Supplement: S2 Fig — Probe density declines with eccentricity on both bottom-up (A) and top-down (B) maps (see overall decreasing characteristic of plots); in both cases, there is little difference between poor and rich insertions (open/solid in A, red/green in B; smooth lines show polynomial 2-degree fits), although there appears to be a moderate trend for rich insertions to exceed poor insertions near the fovea, and poor insertions to exceed rich insertions at 6–8 degrees of eccentricity (see blue trace plotting rich/poor log-ratios; shading shows ±1 SEM). Human sensitivity also declines with eccentricity as expected [109] (overall decreasing characteristic in C-D), but it displays different trends for poor/rich differential effects: no difference between poor and rich insertions on the bottom-up map at any eccentricity (C), and clear differences on the top-down map at all eccentricities (D). Error bars show ±1 SEM. (TIF) [file pbio.1002611.s003.tif]

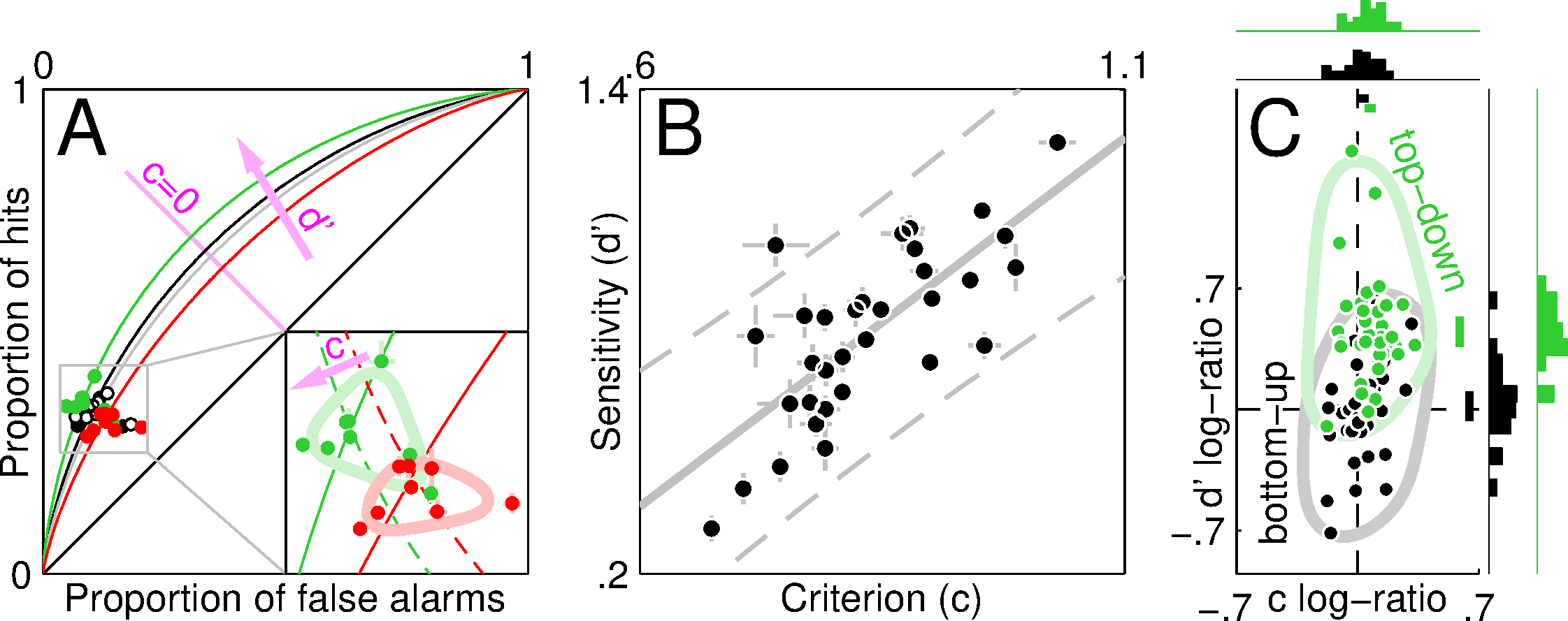

Supplement: S3 Fig — A shows ROC plot [35] of individual data (1 symbol per observer) pooled across conditions that showed a top-down effect without bottom-up effect, for bottom-up poor/rich (black open/solid) and top-down poor/rich (red/green) insertions. Solid lines show best-fits of equal-variance SDT model for variations of sensitivity (d′), dashed lines in inset to A show fits for variations of criterion c; gray/black lines refer to bottom-up poor/rich data, red/green to top-down poor/rich data. Inset magnifies top-down rich/poor data clusters with associated d′/c fits. B plots d′ against c computed under the equal-variance assumption for all data points in A; the 2 quantities are clearly correlated. Error bars show ±1 SEM. Solid line shows best linear fit, dashed lines show 95% confidence intervals for fit. C plots rich/poor log-ratios computed from both d′ (y axis) and c (x axis) with reference to bottom-up (black) and top-down (green) maps; segments near x/y axes show 95% confidence intervals around mean values and demonstrate that the top-down fractional effect for sensitivity (green vertical segment near right y axis) is much greater than the effect for criterion shifts (green horizontal segment near top x axis). Axes in C have been scaled to match for direct comparison. ROC, receiver operating characteristic. (TIF) [file pbio.1002611.s004.tif]
